# Perfluorooctanoic acid disrupts hepatic metabolism in the developing chicken embryo

**DOI:** 10.1007/s11306-025-02374-5

**Published:** 2025-12-01

**Authors:** Nikolai Scherbak, Daniel Duberg, Matilda Kråkström, Pauli Tikka, Alex M. Dickens, Magnus Engwall, Matej Orešič, Tuulia Hyötyläinen

**Affiliations:** 1 MTM Research Centre, School of Science and Technology, Örebro University, SE-701 82 Örebro, Sweden; 2 Turku Centre for Biotechnology, University of Turku and Åbo Akademi University, FI-20520 Turku, Finland; 3 Department of Chemistry, University of Turku, 20500 Turku, Finland; 4 School of Medical Sciences, Örebro University, SE-701 82 Örebro, Sweden; 5 Department of Life Technologies, University of Turku, FI-20014 Turku, Finland

**Keywords:** Avian model, Bile acids, Liver metabolism, Lipids, Mass spectrometry, Perfluorooctanoic acid

## Abstract

**Introduction:**

Perfluorooctanoic acid (PFOA) is a widespread environmental contaminant that interferes with multiple biological pathways, with lipid metabolism being particularly vulnerable. Early-life exposure may disrupt hepatic function during development, but the underlying mechanisms are not fully understood.

**Objectives:**

This study investigated how *in ovo* exposure to PFOA affects hepatic metabolism in the developing chicken embryo, with a focus on identifying altered metabolic pathways and potential mediators of toxicity.

**Methods:**

Fertilized chicken eggs (*Gallus gallus domesticus*) were exposed *in ovo* to six concentrations of PFOA (0–5 µg/g egg). Embryonic liver tissues were analysed by comprehensive metabolomic profiling using two complementary ultra-high-performance liquid chromatography–quadrupole time-of-flight mass spectrometry (UHPLC-QTOF-MS) platforms.

**Results:**

We identified 499 metabolites, including lipids, bile acids, carboxylic acids, amino acids, and phenolic compounds. PFOA exposure caused dose-dependent disturbances in lipid, bile acid, and amino acid metabolism. Notably, multiple secondary bile acids were detected and found to be strongly affected by PFOA, suggesting a central role of bile acid modulation in mediating its effects.

**Conclusions:**

*In ovo* exposure to PFOA disrupts hepatic metabolism in developing chicken embryos, particularly through alterations in bile acid, lipid, and amino acid pathways. These metabolic changes may impair energy production, endocrine regulation, and organ development, with possible long-term health consequences.

**Supplementary Information:**

The online version contains supplementary material available at 10.1007/s11306-025-02374-5.

## Introduction

Per- and polyfluoroalkyl substances (PFAS), including perfluorooctanoic acid (PFOA), are widely applied in various industrial sectors such as in food packaging, carpet manufacturing, firefighting foam production, and textiles. Despite the phasing out of PFOA and perfluorooctanesulfonic acid (PFOS) in several countries, their presence endures in the environment due to their remarkable resistance to degradation over time. Additionally, certain newer PFAS degrade into PFOA and PFOS, further contributing to their persistence (Kolanczyk et al. 2023). Due to their persistent nature and potential for bioaccumulation, both PFOS and PFOA are thus still ubiquitous in our environment and give rise to concerns about chronic low-level exposure and its cumulative impact on human health. Epidemiological studies identified associations between PFOA exposure and increased risks of thyroid dysfunction, developmental abnormalities, impairment of lipid and glucose metabolism, and increased blood pressure(Cardenas et al. 2017, He et al. 2018, Sun et al. 2018, Alderete et al. 2019, Marks et al. 2019, Liu et al. 2023). Dysregulation of lipid and glucose metabolism can, in turn, contribute to the development of metabolic disorders such as obesity, type 2 diabetes, and dyslipidemia.

Certain well-studied PFAS — particularly long-chain perfluoroalkyl acids such as PFOA and PFOS — have been shown to disrupt multiple metabolic pathways, including lipid, energy, amino acid, cholesterol and bile acid metabolism. (Guo et al. 2022, India-Aldana et al. 2023). However, PFAS constitute a highly heterogeneous chemical class, and these effects cannot be assumed to apply uniformly across all PFAS structures. The disruption of lipid metabolism by PFAS has been most consistently demonstrated for long-chain legacy PFAS such as PFOA and PFOS, which have been shown to inhibit mitochondrial fatty acid β-oxidation(Geng, Musse et al. 2019), activate PPARα, and interfere with bile acid–regulated farnesoid X receptor (FXR) signaling (Bjork et al. 2011, Behr et al. 2020). Emerging evidence also suggests similar effects for PFHxS (perfluorohexane sulfonate) and, to a lesser extent, PFNA and PFDA, while ultra-short-chain and most novel fluorinated alternatives have not demonstrated these effects consistently Several PFAS can also activate the hepatocyte nuclear factor 4 (HNF4), a pivotal regulator of transcription in lipid and amino acid metabolism (Han et al. 2017). Recent studies, using PPARα^−/−^ mice suggest also that PFOA can induce liver toxicity *via* PPARα-independent mechanisms (Filgo et al. 2015) and that these effects may be mediated *in utero* (Quist et al. 2015). Indeed, developmental exposure to PFOA has also been linked to weight gain, decreased serum leptin, and reduced insulin sensitivity later in life (Hines et al. 2009). The complex network of metabolic pathways perturbed by various PFAS highlights the liver’s crucial role as the primary metabolic organ regulating lipid and steroid metabolism. Interestingly, these impacts can be initiated as early as *in utero*. Our recent findings in human and experimental models support the hypothesis that many of these classical PFAS-associated changes may be mediated by the bile acids (McGlinchey et al. 2019, Sinisalu et al. 2021, Sinioja et al. 2022), which are important regulators of hepatic lipid and glucose homeostasis and energy metabolism. It is also important to note that the bile acid metabolism during fetal development is substantially different from that in postnatal life (Setchell et al. 1988, Macias et al. 2009, Seki et al. 2011).

While epidemiological studies provide insights into the human health impact, experimental models are indispensable for probing into the underlying mechanisms driving these observed effects. Both i*n vitro* and in vivo models are commonly applied in exposure studies. However, in vitro studies fall short of capturing complex interactions among different organs or cellular compartments, such as those inherent to bile acid metabolism, which encompasses tightly regulated enterohepatic circulation, and interaction through the gut-liver axis. In studies concerning early-life exposures, the use of avian egg injection methods presents numerous advantages. These methods offer direct prenatal exposure, minimal maintenance requirements, and precise control over dosage and exposure conditions due to the contained and uniform nature of the egg (Farhat et al. 2013, Nguyen et al. 2022). The egg’s self-containment and uniform size allows accurate control of the dose and exposure of the test substance and most of the significant confounding factors (e.g., maternal toxicity, maternal care, litter effects) are absent. Moreover, a single dose is sufficient as the chemical will not be excreted from the egg. Avian *in ovo* exposure models have been used for investigation of exposure to pesticides and polycyclic aromatic hydrocarbons (Franci et al. 2018, Nisha et al. 2018), PFAS (Geng et al. 2019), flame retardants (Guigueno et al. 2019, Nguyen et al. 2022), chlorinated paraffins (Fernie et al. 2020) and methylmercury (Yu et al. 2017, Hanas et al. 2020). Specifically, the PFAS exposure model showed that exposure triggered changes in hepatic lipid metabolism, particularly in fatty acid beta oxidation (Geng et al. 2019).

PFAS have been detected in eggs from both commercial laying hens and wild bird populations(Göckener et al. 2020, Gazzotti et al. 2021, Ricolfi et al. 2024). In an Italian monitoring study, PFOS, PFOA, PFNA and PFHxS were found in 54–74% of tested egg samples, with PFOS levels reaching up to 3.5 µg/kg(Gazzotti et al. 2021). recent meta-analysis of PFAS uptake and tissue distribution in wild birds confirmed that liver and egg yolk are primary accumulation sites (Ricolfi et al. 2024). Across global wildlife studies, PFOS consistently emerges as the dominant PFAS, especially in predatory, fish-eating and coastal species such as gulls, ospreys and eagles, with both liver and eggs frequently showing the highest burdens (Lopez-Antia et al. 2017, Kesic et al. 2023).In this study, we investigated the impact of PFOA exposure on hepatic metabolism using an avian *in ovo* exposure model. Embryos were exposed to a wide range of PFOA concentrations, all selected below the threshold for acute toxicity. The primary aim was to assess subtoxic, environmentally relevant exposure levels, reflecting real-world conditions rather than extreme toxicological scenarios. Specifically, we focused on early developmental effects that may not result in overt toxicity but could induce subtle metabolic reprogramming or latent health risks later in life. To capture these effects, we conducted comprehensive metabolic profiling combined with a targeted PCR array to examine key genes involved in lipid metabolism.

## Materials and methods

### Chemicals

All solvents were HPLC grade or LC-MS grade, from Honeywell (Morris Plains, NJ, USA), Fisher Scientific (Waltham, MA, USA) or Sigma-Aldrich (St. Louis, MO, USA). Mass spectrometry grade ammonium acetate and reagent grade formic acid were also from Sigma-Aldrich (St. Louis, MO, USA). The calibration and internal standards are listed in the Supplementary material, with abbreviations given. For quality assurance (QA), standard reference material serum SRM 1950 (for lipidomics and metabolomics) and 1957 (for PFAS and bile acids) was purchased from the National Institute of Standards and Technology (NIST) at the US Department of Commerce (Washington, DC, USA). All glassware and analytical syringes used were thoroughly rinsed with methanol (three times).

### *In Ovo* exposure

Fertilized chicken eggs were purchased from Ova Productions (Vittinge, Sweden) and kept at 10–12 °C until incubation. The exposure was done as described in our earlier study (Nordén et al. 2012). In brief, the chicken eggs were randomly divided to control and dose groups, and they were injected on day 4 of incubation. The experimental protocol was approved by the Swedish Animal Welfare Agency.

The PFOA solutions were prepared by dissolving PFOA (Chemica ≥ 98%, Lot 77282, about 21% branched isomer) in dimethyl sulfoxide (DMSO). The solution was further diluted with water to yield five concentrations of PFOA, both with the same final concentration of DMSO (5%, v/v). A water solution with 5% DMSO (v/v) was used as control.

The 1 µL injection of either solvent or the PFOA was done through a hole drilled in the shell above the air pocket. The dose groups are shown in Table 1, with each dose group having five replicates. Holes were sealed and eggs were incubated (37.5 °C, 60% humidity) and were turned in a six-hour cycle.

Chicken embryos were sacrificed and dissected on day 19 of incubation after the exposure.

**Table 1:** **PFOA **expo**s**ure concentrations and concentrations measured in the liver of chicken embryos, with five replicates/dose, with the dose and PFOA measured in the liver. The level of PFOA and other PFAS tested (PFOS, PFDA, PFHxS, PFuNDA) in control eggs and solvent controls were below level of quantification

PFOA exposure group	PFOA dose µg/g	Measured PFOA (µg/g)
Ctrl	Non treated	0.00
Solvent control	DMSO	0.00
1	0.16	0.17
2	0.50	0.72
3	1.60	2.02
4	5.00	3.40
5	10.00	10.54

### LC-MS analysis

All samples were randomized before sample preparation and analysis. Liver samples were weighted, and phosphate-buffered saline (PBS) was added so that the ratio of tissue to buffer was 1 mg tissue to 10 µL buffer and the samples were homogenized manually. Three extraction methods described below were applied, the first one for extraction of bile acids, PFAS and other semipolar compounds, the second one for the extraction of lipids and the third one for steroid hormones. Three methods were used for the analysis (Supplementary Table 1), namely one method for comprehensive profiling of polar and semipolar metabolites and PFOA, one method for comprehensive profiling of lipids, using an ultra-high-performance liquid chromatography quadrupole time-of-flight mass spectrometry (UHPLC-QTOFMS) and the third method for target analysis of steroids (Supplementary Table 2). Briefly, the UHPLC system used in this work was a 1290 Infinity II system from Agilent Technologies (Santa Clara, CA, USA). The system was equipped with a multi sampler (maintained at 10 °C), a quaternary solvent manager and a column thermostat (maintained at 50 °C), and the QTOFMS was equipped with dual ESI ionization source. For the steroid analysis, a Sciex Exion LC system equipped with two binary pumps, an autosampler (maintained at 10 °C) and a column oven (maintained at 35 °C) was connected to a Sciex 7500 triple quadrupole mass spectrometer. SciexOS 3.1 was used data acquisition (Table 2). MassHunter B.06.01 (Agilent).

**Table 2:** Integrated pathway analysis of metabolomics and transcriptomics data. Results shown for the exposure concentration 0.1 µg/mg exposure. At higher concentrations, same pathways were dysregulated in transcriptomics, although some of them did not reach statistical significance

Pathway	Total	Hits	Raw *p*	FDR	Impact
Valine, leucine and isoleucine degradation	86	13	3.97E-14	5.90E-12	1.03
PPAR signaling pathway	69	12	7.19E-14	5.90E-12	0.47
Fatty acid degradation	83	11	1.94E-11	1.06E-09	2.84
Butanoate metabolism	66	10	4.36E-11	1.79E-09	0.87
Aminoacyl-tRNA biosynthesis	95	9	3.14E-08	1.03E-06	0.12
Propanoate metabolism	80	8	1.28E-07	3.49E-06	0.57
Glyoxylate and dicarboxylate metabolism	93	8	4.15E-07	9.72E-06	0.20
Synthesis and degradation of ketone bodies	16	4	5.06E-06	9.22E-05	2.00
Adipocytokine signaling pathway	71	6	1.43E-05	2.35E-04	0.27
Lysine degradation	109	6	1.62E-04	2.42E-03	0.22
Apelin signaling pathway	128	6	3.89E-04	4.90E-03	0.13
Valine, leucine and isoleucine biosynthesis	26	3	9.49E-04	1.08E-02	0.12
beta-Alanine metabolism	59	4	9.92E-04	1.08E-02	0.26
Pyruvate metabolism	63	4	1.27E-03	1.30E-02	0.33
Tryptophan metabolism	121	5	2.14E-03	2.06E-02	0.12
Insulin signaling pathway	125	5	2.47E-03	2.25E-02	0.03
FoxO signaling pathway	127	5	2.64E-03	2.28E-02	0.04
Glycine, serine and threonine metabolism	87	4	4.15E-03	3.40E-02	0.19
Tight junction	157	5	6.52E-03	5.09E-02	0.02
Primary bile acid biosynthesis	62	3	1.14E-02	8.12E-02	0.12
Alanine, aspartate and glutamate metabolism	62	3	1.14E-02	8.12E-02	0.32
Fatty acid elongation	65	3	1.29E-02	8.84E-02	1.57

#### Analysis of polar and semipolar metabolites and PFOA

For analysis of bile acids, PFAS and polar metabolites, a combined target-non-target method for the analysis of semipolar metabolites and pollutants. 50 µl of liver homogenate was extracted with 400µL of cold MeOH/H_2_O containing the internal standard mixture (Heptadecanoic acid, Lactic acid-d3, Tryptophan-d5, 1-D4-cholic acid, 1-D4-deoxycholic acid, 1-D4- chenodeoxycholic acid,1-D4-glyco cholic acid,1-D4-glycochenodeoxycholic acid,1-D4-glycolithocholic acid,1-D4-glycoursocholic acid,1-D4-lithocholic acid, 1-D4-taurocholic acid, 1-D4-ursocholic acid, PFOA-13C8, PFNA-13C5, PFUndA-13C7, PFHxS-13C3 and PFOS-13C8). The tube was vortexed and ultrasonicated for 3 min, followed by centrifugation (10000 rpm, 5 min). After centrifuging, 350 µl of the upper layer of the solution was transferred to the LC vial and evaporated under the nitrogen gas to the dryness. After drying, the sample was reconstituted into 60 µl of MeOH: H2O (70:30).

Quantitation was done using 6-point calibration (PFOA C = 3.75–120 ng/mL, bile acids c = 20–640 ng/mL, polar metabolites c = 0.1 to 80 µg/mL). Quantification of other bile acids was done using the following compounds: CDCA, CA, DCA, GCDCA, GCA, GDCA, GDCA, GHCA, GHDCA, GLCA, GUDCA, HCA, HDCA, LCA, αMCA, T-α-MCA, T-β-MCA, TCDCA, TCA, THCA, TDCA, THDCA, TLCA, TωMCA and TDCA and polar metabolites was done using alanine, citric acid, fumaric acid, glutamic acid, glycine, lactic acid, malic acid, 2-hydroxybutyric acid, 3-hydroxybutyric acid, linoleic acid, oleic acid, palmitic acid, stearic acid, cholesterol, fructose, glutamine, indole-3-propionic acid, isoleucine, leucine, proline, succinic acid, valine, asparagine, aspartic acid, arachidonic acid, glycerol-3-phosphate, lysine, methionine, ornithine, phenylalanine, serine and threonine. In addition to the measurement of only PFOA, we additionally analyzed Perfluorobutanoic acid, Perfluorobutane sulfonate, Perfluorodecanoic acid, Perfluorododecanoic acid, Perfluorododecane sulfonate, Perfluorodecane sulfonate, Potassium Perfluoro-4-ethylcyclohexanesulfonate, Perfluoroheptanoic acid, Perfluoroheptane sulfonate, Perfluorohexane sulfonate, Perfluorononanoic acid, Perfluorononane sulfonate, Perfluorooctane sulfonamide, Perfluoropentanoic acid, Perfluoro pentane sulfonate, Perfluorotetradecanoic acid, Perfluorotridecanoic acid and Perfluoroundecanoic acid. To ensure PFAS free experiments, we have performed blank extractions and measured PFAS or any other contamination during our analytical procedure. The levels in all PFAS were below the detection limits.

Standard solutions extracted blanks (*n* = 3), pooled QC samples (*n* = 3, an aliquot of each sample pooled), in-house serum QC and NIST CRM 1950 (human plasma) were analyzed together with the samples for quality control. Identification was done based on in-house library (m/z, MS/MS, retention times) that is based on analysis of authentic standards. The PFOA concentrations measured in the NIST CRM were in good agreement with the measured PFOA concentrations, with variation < 11% of the certified values.

#### Lipidomics

For lipidomics the samples were extracted using a modified version of the previously-published Folch procedure (Sen et al. 2022). In short, 10 µL of 0.9% NaCl and, 120 µL of CHCl3: MeOH (2:1, v/v) containing the internal standards (c = 2.5 µg/mL) was added to 10 µL of sample homogenate. The standard solution contained the following compounds: PE(17:0/17:0), SM(d18:1/17:0), NCer(d18:1/17:0), PC(17:0/17:0), 1LPC(17:0),CE(17:0),PC(16:0/d31/18:1), and, TG(17:0/17:0/17:0). The samples were vortex mixed and incubated on ice for 30 min after which they were centrifuged (9400 × g, 3 min). 60 µL from the lower layer of each sample was then transferred to a glass vial with an insert and 60 µL of CHCl3: MeOH (2:1, v/v) was added to each sample. The samples were stored at −80 °C until analysis.

Calibration curves using 1-hexadecyl-2-(9Z-octadecenoyl)-sn-glycero-3-phosphocholine (PC(16:0e/18:1(9Z))), 1-(1Z-octadecenyl)−2-(9Z-octadecenoyl)-sn-glycero-3-phosphocholine (PC(18:0p/18:1(9Z))), 1-stearoyl-2-hydroxy-sn-glycero-3-phosphocholine (LPC(18:0)), 1-oleoyl-2-hydroxy-sn-glycero-3-phosphocholine (LPC(18:1)), 1-palmitoyl-2-oleoyl-sn-glycero-3-phosphoethanolamine (PE(16:0/18:1)), 1-(1Z-octadecenyl)−2-docosahexaenoyl-sn-glycero-3-phosphocholine (PC(18:0p/22:6)) and 1-stearoyl-2-linoleoyl-sn-glycerol (DG(18:0/18:2)), 1-(9Z-octadecenoyl)-sn-glycero-3-phosphoethanolamine (LPE(18:1)), N-(9Z-octadecenoyl)-sphinganine (Cer(d18:0/18:1(9Z))), 1-hexadecyl-2-(9Z-octadecenoyl)-sn-glycero-3-phosphoethanolamine (PE(16:0/18:1)) from Avanti Polar Lipids, 1-Palmitoyl-2-Hydroxy-sn-Glycero-3-Phosphatidylcholine (LPC(16:0)), 1,2,3 trihexadecanoalglycerol (TG(16:0/16:0/16:0)), 1,2,3-trioctadecanoylglycerol (TG(18:0/18:0/18:)) and 3β-hydroxy-5-cholestene-3-stearate (ChoE(18:0)), 3β-Hydroxy-5-cholestene-3-linoleate (ChoE(18:2)) from Larodan, were prepared to the following concentration levels: 100, 500, 1000, 1500, 2000 and 5000 ng/mL (in CHCl3:MeOH, 2:1, v/v) including 1250 ng/mL of each internal standard.

Standard solutions, extracted blanks (*n* = 3), pooled QC samples (*n* = 3, an aliquot of each sample pooled), in-house serum QC and NIST CRM 1950 (human plasma) were analyzed together with the samples for quality control. Identification was done based on in-house library (mz, MS/MS, retention times).

#### Data preprocessing for metabolomics and lipidomics

Mass spectrometry data processing was performed using the open-source software package MZmine 2.53 (Pluskal et al. 2010). The following steps were applied: (1) Crop filtering with a m/z range of 350–1200 m/z and an RT range of 2.0 to 12 min, (2) Mass detection with a noise level of 750, (3) Chromatogram builder with a minimum time span of 0.08 min, minimum height of 1000 and a m/z tolerance of 0.006 m/z or 7.0 ppm, (4) Chromatogram deconvolution using the local minimum search algorithm with a 70% chromatographic threshold, 0.05 min minimum RT range, 5% minimum relative height, 1200 minimum absolute height, a minimum ration of peak top/edge of 1.2 and a peak duration range of 0.08–5.0, (5) Isotopic peak grouper with a m/z tolerance of 5.0 ppm, RT tolerance of 0.05 min, maximum charge of 2 and with the most intense isotope set as the representative isotope, (6) Join aligner with a m/z tolerance of 0.009 or 10.0 ppm and a weight for of 2, a RT tolerance of 0.1 min and a weight of 1 and with no requirement of charge state or ID and no comparison of isotope pattern, (7) Peak list row filter with a minimum of 10% of the samples, (8) Gap filling using the same RT and m/z range gap filler algorithm with an m/z tolerance of 0.009 m/z or 11.0 ppm, (9) Identification of lipids using a custom database search with an m/z tolerance of 0.009 m/z or 10.0 ppm and a RT tolerance of 0.1 min, and (10) Normalization using internal standards: Lipids: PE(17:0/17:0), SM(d18:1/17:0), Cer(d18:1/17:0), LPC(17:0), TG(17:0/17:0/17:0) and PC(16:0/d30/18:1)) for identified lipids and closest ISTD for the unknown lipids followed by calculation of the concentrations based on lipid-class concentration curves. For polar and semipolar metabolites, the following ISTDs were used Valine-d8, Glutamic acid-d5, Succinic acid-d4, Heptadecanoic acid, Lactic acid-d3, Citric acid-d4. 3-Hydroxybutyric acid-d4, Arginine-d7, Tryptophan-d5, Glutamine-d5, 1-D4-CA,1-D4-CDCA,1-D4-CDCA,1-D4-GCA,1-D4-GCDCA,1-D4-GLCA,1-D4-GUDCA,1-D4-LCA,1-D4-TCA, 1-D4-UDCA.

For data filtering, we removed compounds that were present at blank samples (peak area < 5 times that of blank) and compounds that had RSD > 30% in the pooled quality control samples. MS/MS data was done for the pooled quality control samples using auto MS/MS mode.

#### QC/QA for metabolomics and lipidomic

To evaluate the robustness of the method, relative standard deviations were calculated for the pooled samples, both for lipidomics as well as for polar metabolites. The pooled samples were prepared by taking an aliquot (10 µl) of each liver extract, separately for lipidomic and polar metabolite methods, and pooling them, and aliquoting the pool into three separate vials. For lipidomics, the RSD was on average 10.8% for identified metabolites and 14.6% for all compounds and of the identified compounds, 55% had a RSD of < 10%. For polar and semipolar metabolites, the RSD was on average 9.1% for identified metabolites and 13.3% for all compounds and of the identified compounds, 67% had RSD of < 10%. The recoveries of internal standards were on average 84% (Supplementary Table 3).

#### Targeted steroid analysis

For analysis of steroids, liver homogenate (150 µL) was transferred to an eppendorf tube. Internal standard mix (10 µL, containing 2–200 nM 11-keto-testosterone-d3, keto-dihydrotestosterone-d3, Testosterone-d3, Aldosterone-d7, Dihydrotestosterone-d4, Androsterone-d4, 11β-OH-androstenedione-d4, 17α-OH-Pregnenolone-d3, Corticosterone-d8, Pregnenolone-d4, 11-deoxy-cortisol-d7, 11-deoxy-corticosterone-d7, Cortisol-d4, Progesterone-d9, Cortisone-d8, 17α-OH-progesterone-d8, 17β-estradiol-d4, Estrone-d4, Dehydroepiandrosterone-d2 and Androstendione-d3 in acetonitrile: water 30:70) was added. Samples were vortexed and 1 mL metyl-tert-buytl ether was added. Samples were vortexed for 10 min. A portion of the sample (800–900 µL) was transferred to a protein participation filter plate (Supelco Bellefonte, Pennsylvania, USA). The samples were filtered under vacuum, dried under a gentle stream of nitrogen and reconstituted in 50 µL of 30% acetonitrile in water. Calibration curves were prepared for 11β-OH-androstenedione, 11-keto-dihydrotestosterone, 11-keto-testosterone, Androsterone, 11-deoxy-corticosterone, 17α-OH-progesterone, Adrenosterone, Aldosterone, Androstenedione, Corticosterone, Cortisol, Cortisone, Dehydroepiandrosterone, Dihydrotestosterone, 17β-estradiol, Estrone, Progesterone, Testosterone/Epi-testosterone, 11-deoxy-cortisol and 17α-OH-pregnenolone by transferring 20 µL of standard dilution (concentration between 0.025 and 1000 nM) to a vial and adding 40 µL internal standard mix and 20 µL of 30% acetonitrile in water. Standard solutions, extracted blanks (*n* = 3) and pooled QC samples (*n* = 3, an aliquot of each sample pooled) were analyzed together with the samples for quality control. Quantification was performed in SciexOS 3.1 using the internal standard method. Limits of quantitation (LOQ) are given in Supplementary Table 2.

### Focused PCR array

A focused PCR array was applied to analyze 98 genes associated with lipid metabolism. Ca 15 mg of chicken embryo liver tissue exposed either to 0.1–1.0 µg/mg of egg was used for RNA purification using RNeasy^®^ mini kit (QIAGEN, Hilden, Germany) according to manufacturer protocol. Using qPCR, samples were analyzed using Chicken Fatty Acid CTRLMetabolism RT2 profiler PCR array^®^ (Catalogue PAGG-007Z; QIAGEN). These arrays come in 96-well plate format, which include 84 wells containing primers for genes of interest, 5 wells containing housekeeping genes suggested by QIAGEN, and additional controls to analyze genomic DNA contamination, reverse transcription efficiency and PCR array reproducibility. One plate was used to analyze each biological sample, and an electronic pipetting system was used to limit any variation caused by pipetting technique. The qPCR program was set to 40 cycles consisting of the following temperatures and time intervals: an initial denaturation at 95 °C for 5 min, followed by 15 s at 95 and 60 °C for 1 min for 40 cycles using an Applied Biosystems^®^ 9700 thermocycler (Applied Biosystems, Carlsbad, CA). Each run was completed with melting curve analysis to confirm a single amplified product.

### Data analysis

All statistical analyses were performed at the individual lipid and metabolite concentration level as well as at the level of lipid classes. For the latter, individual lipid concentrations in each lipid class were first median normalized, summed, then subsequent data analysis considering each lipid class as a variable was performed. For regression analysis, the individual PFAS concentrations were used as exposure variable. The data were first log transformed and then scaled to zero mean and unit variance. Linear model with covariate adjustments were done using limma method in MetaboAnalyst 5.0 (Chong et al. 2019, Pang et al. 2022). Liver weight was considered as covariate. The p values were corrected for multiple testing using methods false discovery rate (FDR) correction using Significance Analysis of Microarrays and Metabolites (SAM) method. Chord plots and mediation analyses were done with R package 4.3.2 (Team 2017).

Pathway analyses were done by Functional analysis module in MetaboAnalyst 5.0 and for targeted lipids with LIPEA (Acevedo et al. 2018). The Functional analysis module approach supports functional analysis of untargeted metabolomics data generated from HRMS. The pathway analysis was done with the data of the polar and semipolar metabolites, as the pathway analysis for lipidomics data is not sufficiently robust due to the lack of exact structures of the lipids (fatty acid composition, including the position of the double bonds, cis/trans configuration). However, our polar/semipolar panel includes a large number of lipids, except for neutral lipids (CE, DG, TG) that are not covered either by sample preparation nor the negative ion mode. The input data for the pathway analysis comprised complete LC-HRMS data, i.e., both identified and unknown metabolites, obtained in negative ionization mode. First, we performed statistical analyses using t-test between control and each exposure concentration, resulting in fold change, p values and FDR values. The whole input peak list, with peak names given as their numeric mass (m/z) values for putative annotation was used for the pathway analysis. The mass tolerance for the pathway analysis was set at 7 ppm, and we also used advanced option to select representative adducts by removing isotopic adducts as these have been already removed in the data preprocessing step. We applied both Mummichog and Gene set enrichment algorithms with p-value cutoff of 0.05, and utilized the KEGG pathway library for *Gallus gallus* (chicken). In the evaluation of the results, we included only pathways showing minimum 3 significant compound hits, and with either of the algorithms giving a significant pathway match. For joint pathway analysis, using the targeted transcriptomics data and non-target metabolomics data, the same settings were applied.

### Mediation analysis

Mediation analysis was done by using mediate package in R with 1000 simulations (Tingley et al. 2014). The main output of the analysis, the indirect effects of PFOA via bile acids to lipids, was depicted with a Sankey diagram. These indirect effects mean here Average Causal Mediated Effects (ACME), which is the difference between estimated outcome concentrations of lipids when they have or have not been mediated via bile acids (Tingley et al. 2014). The direct effects in the other hand are in this case Average Direct Effects (ADE), which refer to the outcome concentration differences that resulted directly *via* PFOA. ADE can be similar in magnitude and direction as a linear model coefficient value or a correlation coefficient if it does not affect no more than one lipid at a time and during its calculation a simple linear model without related conditions or cofounders can be assumed. Additionally, when simulating both ADE and ACME in the mediate package, two additional measures, a p-value and a proportion of mediated, are calculated. The proportion mediated is calculated for each simulation (here 1,000 times) separately, i.e., ACME/(Total Effect = ACME + ADE), and then takes a median of these values for the final value. The p-value here is a fraction of simulations that are above or below zero, where the lowest one is selected and multiplied with two.

## Results

We investigated the impact of PFOA exposure on metabolism in the chicken embryo liver, using exposure levels below acute toxicity to mimic the environmentally relevant concentrations. The exposure period was selected based on the current recommendations, starting from day 4, and finishing before the hatching. The starting exposure time was selected as four-day-old embryos have passed the first critical period of organogenesis, and lethality due to disturbance from injection decreases and additionally at this time point, it is possible to establish the viability of the egg, an advantage because inclusion of infertile eggs in a study could increase uncontrolled variability (i.e., with day 0 injection.(Heinz et al. 2006, Yu et al. 2025). We did not observe any significant differences in liver weight based on the exposure, and we have previously shown that there were no statistically significant changes in liver somatic index between the exposed groups compared with the control groups (Nordén et al. 2016).

### Metabolomic profile in the chicken embryo liver

**Fig. 1:**
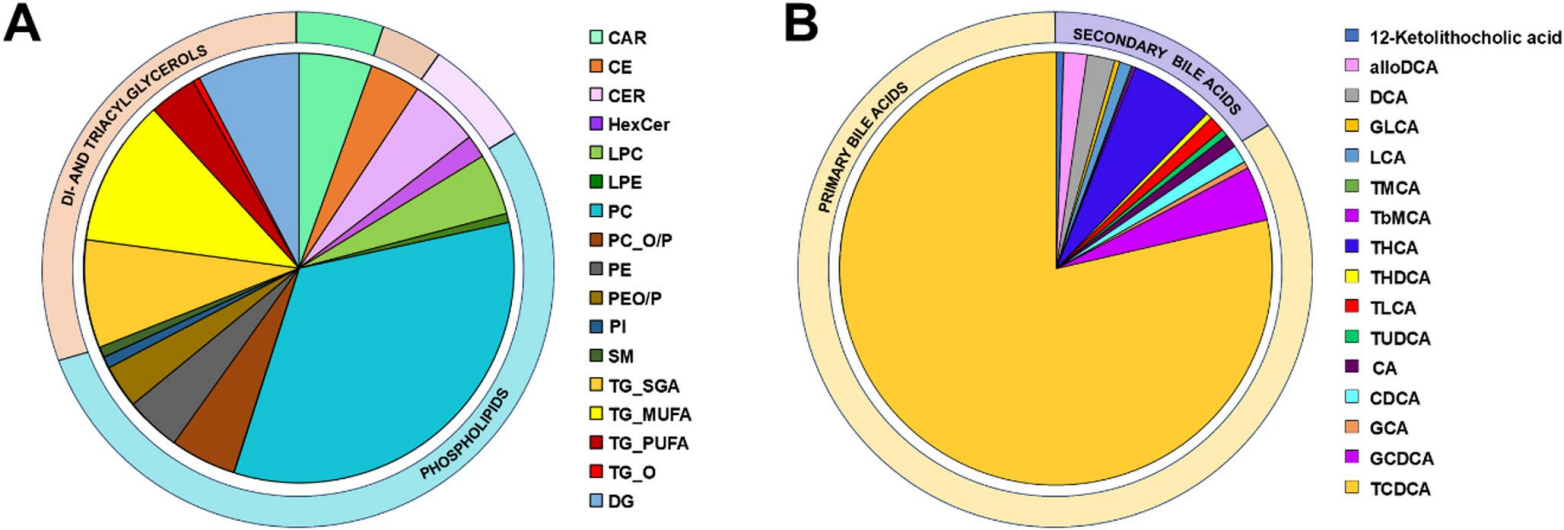
(**A**) Lipid and (**B**) bile acid profiles of control chicken embryo liver

We identified 394 lipids belonging to lipid classes of cholesterol esters (CE), ceramides, lysophosphatidylcholines (LPC), phosphatidylcholines (PC), phosphatidylethanolamines (PE), triacylglycerols (TG), cholesteryl esters (CE), sphingomyelins (SM), phosphatidylinositols (PI), ceramides (Cer), and acylcarnitines (Car). Phospholipids and glycerolipids were the most abundant lipids in the liver. We also identified 105 polar and semipolar metabolites, including bile acids, free fatty acids and other carboxylic acids, amino acids, phenolic metabolites, and others (Fig. 1). The main bile acid in the liver was TCDCA, comprising over 70% of the bile acid pool. We also analyzed the egg white and egg yolk in nonfertilized eggs, to see which bile acids were present. The egg yolk had a significantly higher amount of bile acids, with the major bile acids detected being TCDCA and THCA. Also, several secondary bile acids and their taurine conjugates, such as TLCA, LCA, DCA, TaMCA, TUDCA and TDCA were detected in the nonfertilized eggs.

### Metabolic dysregulation by PFOA

**Fig. 2:**
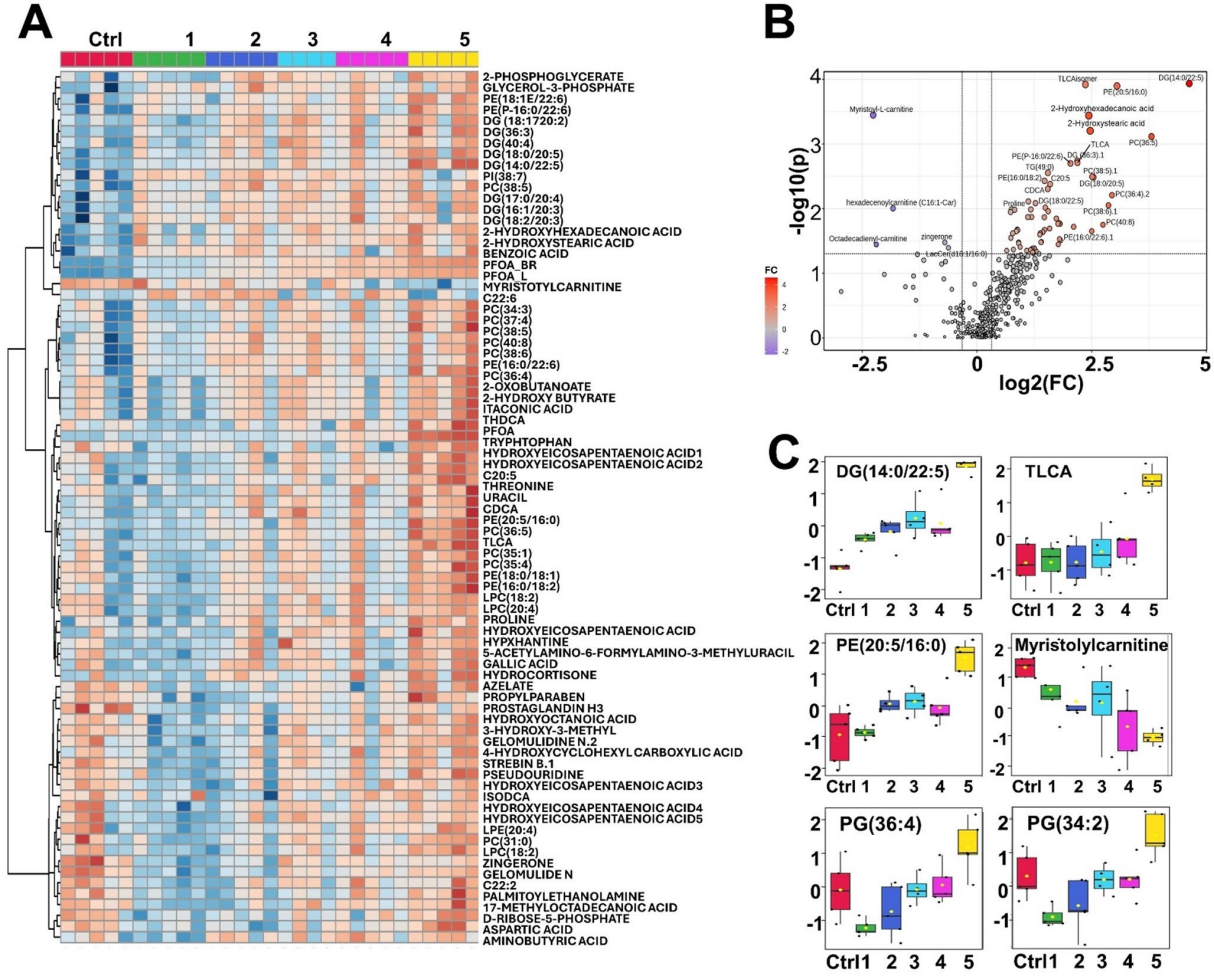
(**A**) Heatmap of lipid and metabolites selected based on ANOVA, (**B**) Highest PFOA exposure vs. control (FC > ± 1.25, *p* > 0.05), and (**C**) examples of metabolites showing significant differences between the study groups

**Fig. 3:**
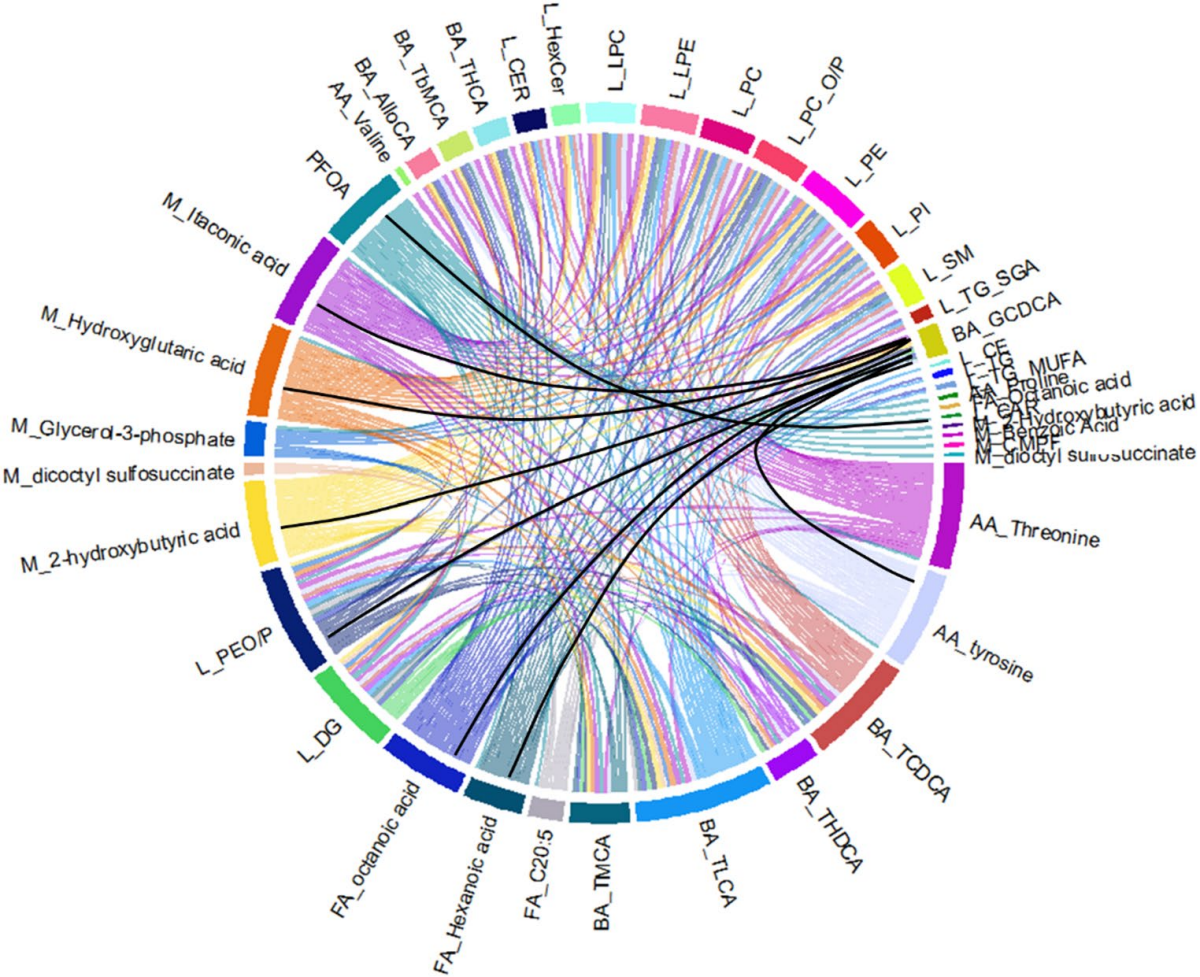
Chord plot of partial correlation analysis between bile acids (BA), amino acids (AA), other polar metabolites (M), lipid classes (L) and PFOA. Intraclass correlations between metabolites and lipid classes are removed. Most of the correlations were positive, the negative correlations are visualized with black lines. For partial correlations, *p* < 0.05, q < 0.1

We observed marked changes in metabolic profiles in the liver after PFOA treatment (Fig. 2), while the PFOA exposure did not have any significant impact on the liver weight (**Supplementary Fig. 1**). DMSO, which was used as a solvent, did not show any significant impact on metabolism. Large number of lipids, amino acids and bile acids were upregulated after PFOA exposure, while three long-chain acylcarnitines and the primary bile acid GCDCA were significantly downregulated (Fig. 2, **Supplementary Table 4**). Particularly DGs and phospholipids (LPC, PC, PE) showed strong upregulation. Partial correlation analysis (Fig. 3) between PFOA, metabolites and lipid classes showed that PFOA was associated mainly with specific bile acids (TCDCA, THDCA, TLCA, TMCA) and polar metabolites (proline, threonine, tyrosine, fatty acids, 2-hydroxybutyric acid, hydroxyglutaric acid, glycerol-3-phosphate, benzoic acid, itaconic acid, CMPF, dioctyl sulfosuccinate) and with four classes of lipids (Car, DG, PE and alkyl-ether PEs). Large number of lipids, especially multiple PGs, displayed nonlinear, U-shaped association with the exposure (Fig. 2C).

### Pathway analysis

**Fig. 4:**
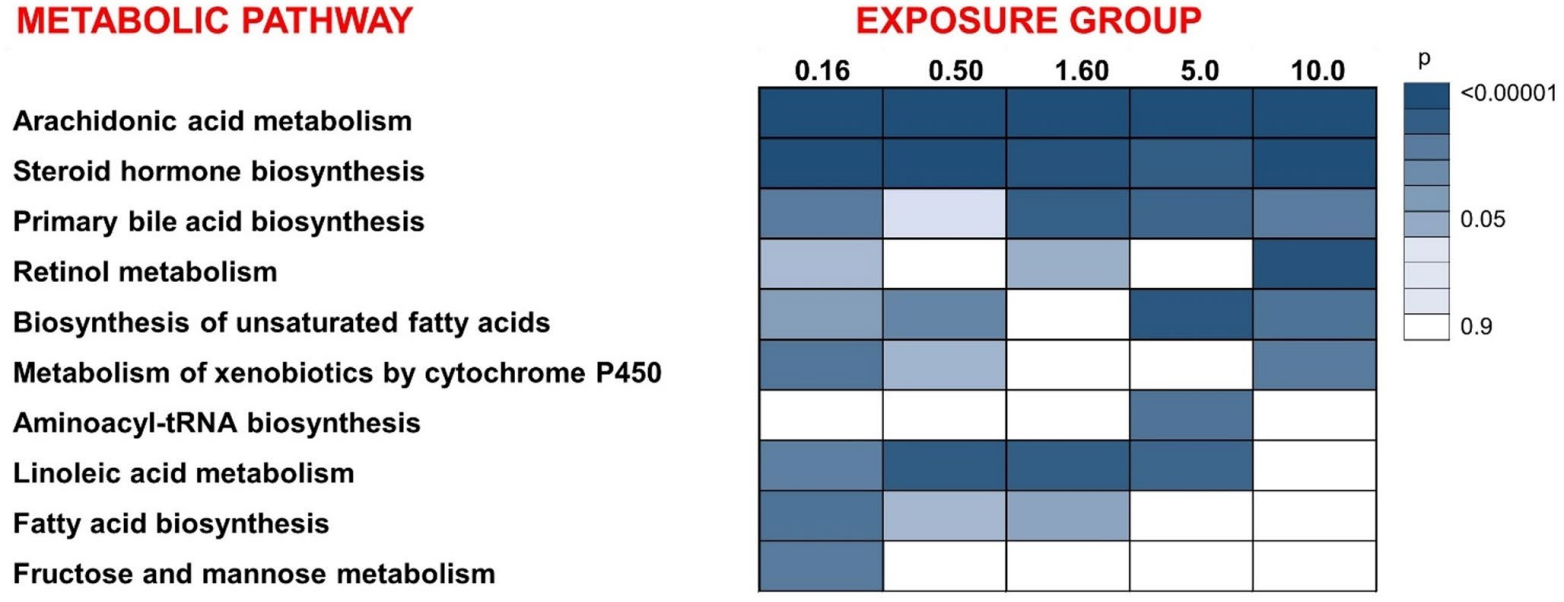
Pathway analysis using both mummichog and gene enrichment analysis algorithms by comparison of each PFOA exposure concentration with control. The p values are visualized by color gradient, from white (not significant) to dark blue

Next, we performed pathway analysis to investigate which metabolic pathways were affected by PFOA exposure (Fig. 4). Because a substantial number of metabolites displayed non-linear dose–response patterns, we performed pathway analysis separately for each exposure concentration, which we found to be a more biologically robust strategy than assuming a monotonic dose–response. The pathway analysis also incorporates putatively annotated features from the non-targeted dataset, including particularly oxylipins related to arachidonic, linoleic and prostaglandin metabolism.

The main metabolic pathways disrupted by the exposure were fatty acid metabolism, bile acid metabolism and steroid hormone biosynthesis, which were dysregulated in majority of the exposure concentrations. We also observed dose-dependent differences in the metabolic responses, for example, at lowest exposure level, we observed significant dysregulation in fructose and mannose metabolism which was not observed at higher exposure levels. Also, the fatty acid biosynthesis was most strongly dysregulated at the three lowest exposure concentrations. The targeted lipid pathway analysis found two significantly altered pathways, namely Glycerophospholipid metabolism and Ferroptosis (*p* < 0.05, FDR < 0.05).

As steroid hormone biosynthesis pathway showed strong dysregulation, we additionally performed targeted steroid analysis. However, due to limited sample amount available, only testosterone + epitestosterone (T + epiT) could be detected in most of the samples (> 92%). T + epiT showed strong positive association with PFOA levels (*R* = 0.56, *p* = 0.0012). When investigating those steroids mapped on the pathway, most detected compounds in the pathway were conjugated and hydroxylated steroids, which could explain why they were not detected with the target panel for free steroid hormones.

Next, we combined our metabolomics data on targeted gene expression data and performed a joint pathway analysis (Table 3). In addition to the pathways obtained in the metabolic pathway analysis, several pathways related to lipid and amino acid metabolism were dysregulated in the joint pathway analysis. Notably, the pathway analysis also indicated significant enrichment of the PPAR signalling pathway, consistent with previous evidence that PFOA can act as a PPAR agonist.

**Table 3:** Integrated pathway analysis of metabolomics and transcriptomics data. Results shown for the exposure concentration 0.1 µg/mg exposure. At higher concentrations, same pathways were dysregulated in transcriptomics, although some of them did not reach statistical significance

Pathway	Total	Hits	Raw *p*	FDR	Impact
Valine, leucine and isoleucine degradation	86	13	3.97E-14	5.90E-12	1.03
PPAR signaling pathway	69	12	7.19E-14	5.90E-12	0.47
Fatty acid degradation	83	11	1.94E-11	1.06E-09	2.84
Butanoate metabolism	66	10	4.36E-11	1.79E-09	0.87
Aminoacyl-tRNA biosynthesis	95	9	3.14E-08	1.03E-06	0.12
Propanoate metabolism	80	8	1.28E-07	3.49E-06	0.57
Glyoxylate and dicarboxylate metabolism	93	8	4.15E-07	9.72E-06	0.20
Synthesis and degradation of ketone bodies	16	4	5.06E-06	9.22E-05	2.00
Adipocytokine signaling pathway	71	6	1.43E-05	2.35E-04	0.27
Lysine degradation	109	6	1.62E-04	2.42E-03	0.22
Apelin signaling pathway	128	6	3.89E-04	4.90E-03	0.13
Valine, leucine and isoleucine biosynthesis	26	3	9.49E-04	1.08E-02	0.12
beta-Alanine metabolism	59	4	9.92E-04	1.08E-02	0.26
Pyruvate metabolism	63	4	1.27E-03	1.30E-02	0.33
Tryptophan metabolism	121	5	2.14E-03	2.06E-02	0.12
Insulin signaling pathway	125	5	2.47E-03	2.25E-02	0.03
FoxO signaling pathway	127	5	2.64E-03	2.28E-02	0.04
Glycine, serine and threonine metabolism	87	4	4.15E-03	3.40E-02	0.19
Tight junction	157	5	6.52E-03	5.09E-02	0.02
Primary bile acid biosynthesis	62	3	1.14E-02	8.12E-02	0.12
Alanine, aspartate and glutamate metabolism	62	3	1.14E-02	8.12E-02	0.32
Fatty acid elongation	65	3	1.29E-02	8.84E-02	1.57

### Bile acid as mediators of the impacts of PFOA exposure on lipids

**Fig. 5:**
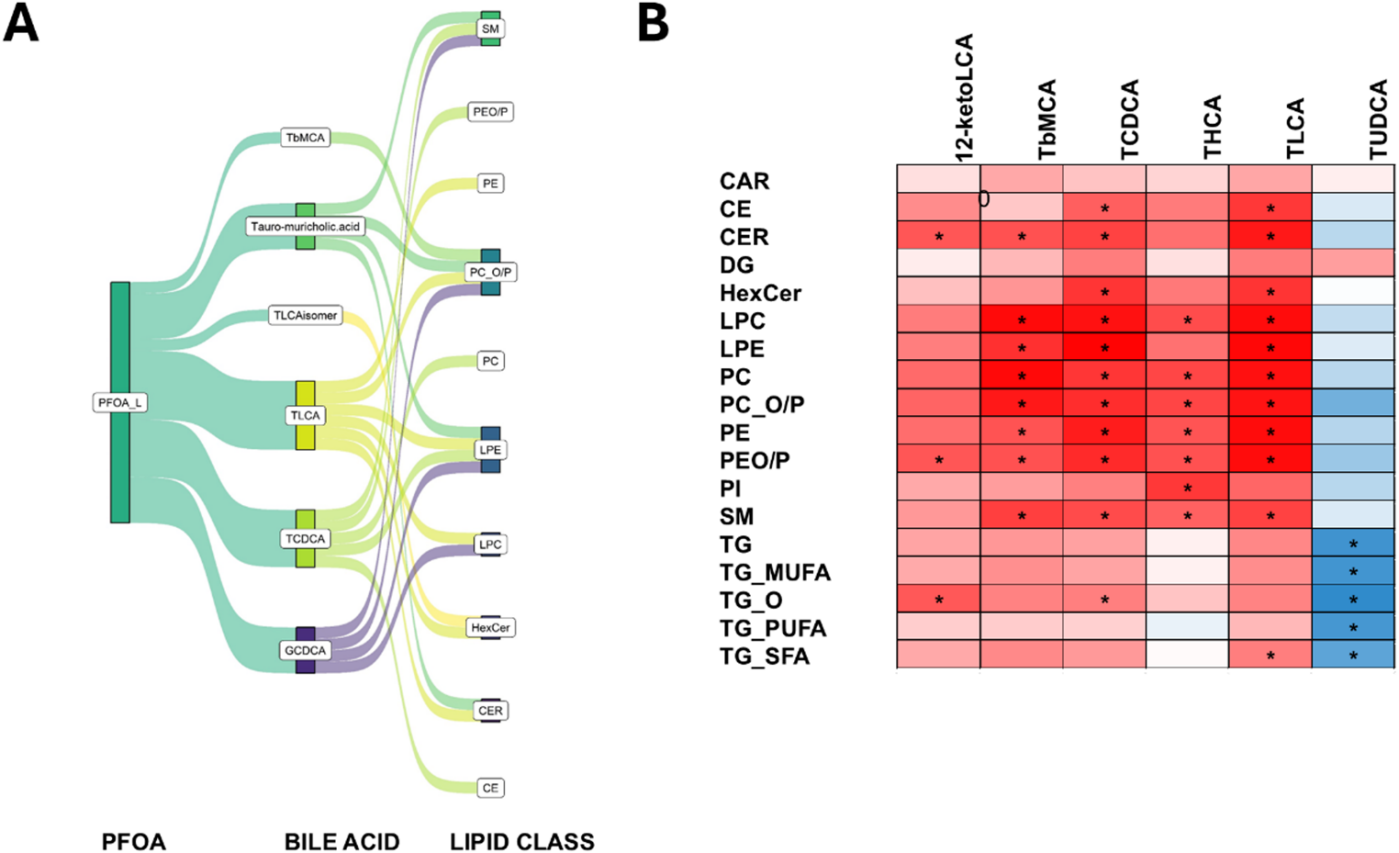
**A** Mediation analysis examining the impact of PFOA exposure on lipids (at lipid class level), with bile acids as potential mediators. The Sankey plot shows selected mediators (bile acids) and lipids (outcomes) for which p-value < 0.05, Average Causal Mediated Effect (ACME) > 0, and proportion mediated > 0.5. Complete results are shown in Supplementary Tables 5 and the outcome and income measures for mediation analysis are explained at methods. **B** Adjusted partial correlations (adjusted with PFOA and liver weight) between bile acids and lipid classes. Only bile acids showing significant correlations are included (*p* < 0.05; q < 0.1)

To further investigate the role of bile acids as potential mediators of the PFOA exposure on lipid metabolism, we performed mediation analysis as well as partial correlation analysis between bile acids and lipid classes, by adjusting the data with the PFOA and liver weight. In mediation analysis, several bile acids were found to mediate the impact of PFOA exposure on hepatic lipids (Fig. 5A). Particularly TLCA was found to mediate the impact on several lipid classes including major phospholipids. In partial correlation analysis (Fig. 5B), taurine conjugated bile acids, particularly LCA, showed a very strong positive correlation with several lipid classes, especially phospholipids. The correlations with TGs were clearly weaker. Interestingly, TUDCA showed an opposite trend, with negative correlations with all classes of TGs. We also performed the partial correlation analysis to the control group only, with very similar results.

## Discussion

We used an avian *in ovo* model to investigate the impact of prenatal PFOA on the liver metabolism in chicken embryo. The concentration range was selected to represent environmentally relevant concentrations, not reaching acute toxicity. We investigated the changes in the level of individual metabolites, and at the pathway level, with pathway level and transcriptomic results capturing non-targeted, systems-level responses, which extend beyond the subset of individually confirmed metabolites.

We observed selective, dose-dependent perturbations in metabolic pathways related to lipid, steroid, bile acid, and fatty acid metabolism. The integration of metabolomics with targeted transcriptomics confirmed dysregulation of lipid metabolic processes and suggested potential involvement of PPAR-related signaling, consistent with prior literature, although causality cannot be definitively established. PPARα is the key regulator of lipid metabolism, involved in regulation of fatty acid uptake and activation, mitochondrial and peroxisomal fatty acid oxidation, ketogenesis, triglyceride turnover, lipid droplet biology, gluconeogenesis and bile synthesis/secretion (Todisco et al. 2022). Our transcriptomic coverage did not include bile acid or steroid-specific receptors, so their regulation may be underrepresented at the transcript level despite clear metabolic evidence. Interestingly, the pathway-level effects did not follow a monotonic dose–response pattern, which is in line with previous literature showing non-linear and often adaptive low-dose responses to PFAS, where early compensatory mechanisms may operate at environmentally relevant exposures but become dysregulated at higher doses (Alijagic et al. 2024).

Of particular interest was the strong dysregulation of arachidonic acid (AA) pathway by PFOA exposure, as this pathway is indicative of oxidative stress, inflammation and immune response. AA derived oxylipins such as prostaglandin E2 and leukotrienes play central roles in immune activation and oxidative stress which, in turn, have been linked with several liver diseases (Sztolsztener et al. 2020, Bovi et al. 2021, Karkucinska-Wieckowska et al. 2022). Interestingly, PPARα can be activated during the inflammatory response by binding polyunsaturated fatty acids and AA-derived oxylipins (Fang et al. 2007, Korbecki et al. 2019), providing a plausible mechanistic link between the observed AA pathway disruption and PPAR-associated signaling. Human studies have also reported PFAS exposure associated changes in arachidonic acid and linoleic metabolism as well as in steroid hormone biosynthesis (Li et al. 2021, India-Aldana, Yao et al. 2023), in agreement with our results. In murine models, PFOA exposure has been linked with changes in arachidonic acid (AA) pathway (Yu et al. 2016) with human studies reporting similar associations (Li et al. 2021).

Bile acid and steroid metabolic pathways were also markedly affected by the exposure. We observed marked upregulation of multiple bile acids, including taurine-conjugated lithocholic acid (TLCA), whereas the primary bile acid GCDCA was reduced. Since embryonic chickens lack a mature gut microbiota, the strong increase in TLCA, classified traditionally as microbially modified bile acid, suggests endogenous hepatic synthesis through alternative developmental pathways (Mitropoulos & Myant 1967, Colombo et al. 1985). While LCA was also detected in egg yolk and in egg white of nonfertilized eggs, the increased (T)LCA must be due to synthesis in the embryonic liver, in the absence of any additional external source. TLCA showed strong associations with phospholipids independent of PFOA concentration, supporting the hypothesis that downstream lipid effects may be indirectly mediated *via* bile acid signaling, rather than by direct PFOA action. Indeed, TLCA is a known agonist of TGR5, a bile acid receptor involved in lipid and energy metabolism (Holter et al. 2020). TLCA synthesis in the liver during embryonic development may indicate a compensatory mechanism independent of gut microbiota, which in adults is typically essential for bile acid metabolism and conjugation. The embryonic liver might prioritize TLCA production to mitigate lipid dysregulation in the absence of microbial involvement (Guo et al. 2016).

Dysregulation of steroid metabolism, as observed in our study, can have a major impact on the fetal development as the steroid hormones are involved in modulating cell differentiation and organ system development during fetal life (Markey et al. 2003) potentially culminating in adverse health impacts later in life, such as cardiometabolic diseases and neuropsychiatric diseases (Bilder et al. 2019, Cardoso & Padmanabhan 2019). Here, we observed a strong positive correlation of testosterone + epitestosterone with PFOA exposure levels. Elevated testosterone levels during the fetal development can have wide-ranging effects on sexual differentiation, brain and behavioral development, physical growth, immunological development and therefore, on future health risks (Auyeung et al. 2009, McCarthy 2016, Dooley et al. 2022).

We also observed selective downregulation of long-chain acylcarnitines, consistent with impaired mitochondrial β-oxidation, which aligns with previous reports of PFAS disrupting fatty acid oxidation (Lu et al. 2019), and increase the expression of hepatic genes involved in fatty acid and triglyceride synthesis, thus potentially prompting steatosis through biasing of the balance between lipogenesis and lipolysis (Das et al. 2017). Conversely, multiple amino acids were significantly upregulated reflecting possible altered nutrient utilization and intensified metabolic demand during embryonic development. (Wu et al. 2017). (Zhao et al. 2023) Furthermore, energy production during fetal development is highly dependent on amino acid metabolism, particularly as oxidative fuel use increases during maternal nutrient deprivation (Liechty 2003). Changes in amino acid and lipid composition could also suggest alteration of gluconeogenesis, as the late term embryonic chicken liver derives > 90% of its energy through the oxidation of yolk lipids while glucose is provided by glycogen stores and gluconeogenesis from amino acids, glycerol, and lactate (Surugihalli, Farley et al. 2022). Our findings are consistent with human and animal studies reporting PFAS-associated amino acid alterations (McGlinchey et al. 2019, Stratakis et al.^ 2020^). Additional markers, such as itaconic acid and hypoxanthine further indicate inflammatory and energetic stress. Increased levels of itaconic acid suggest response to inflammation and it have shown to impair the mitochondrial function (Belosludtsev et al. 2020) and the itaconic acid can also affect cellular metabolism and energy production. The upregulated hypoxanthine, on the other hand, has also been reported to be upregulated in a study of highly PFAS-exposed individuals (Lu, Gao et al. 2019). Hypoxanthine is a degradation product of purine, which is finally oxidized to uric acid. In chicken embryos, hypoxanthine has been suggested to promote myoblast proliferation (Ii et al. 1985).

Overall, the observed alterations in metabolic pathways and signaling networks in the embryo liver suggest potential adverse effects relevant to fetal development, including oxidative stress and inflammation, and potentially disruptions in organogenesis, endocrine function, energy metabolism, and nutrient utilization. The important question is how these findings can be translated into understanding of health impacts in humans. During the prenatal stage, chicken embryos primarily rely on the nutrients stored in the egg yolk for the development, while mammals rely on nutrients delivered through the placenta from the mother. While chickens and mammals share fundamental metabolic pathways, the chickens have higher metabolic rates than mammals and may utilize glucose at different rates. Overall, the knowledge of hepatic metabolism across humans and chicken during embryonic stages is scare (Sato et al. 2008).

Importantly, our present findings are consistent with our previous observations that prenatal and early-life PFAS exposure in non-obese diabetic (NOD) mice (McGlinchey et al. 2019, Sinioja et al. 2022) human fetal liver (Hyötyläinen et al. 2024), and in human hepatocytes (Alijagic et al 2024)), In these studies, we have hypothesized that the fetal liver might be capable of synthesizing LCA even in the absence of gut microbiota although we could not exclude the maternal impact in these studies. The current *in ovo* chicken model, which eliminates maternal and microbial influence, provides strong supporting evidence for this hypothesis. Moreover, in our recent in vitro exposure model using human hepatocytes (HepaR2) (Alijagic et al 2024), FAS exposure also induced robust upregulation and excretion of LCA conjugates, confirming that human liver cells themselves can synthesize and export LCA independently of gut microbiota — potentially as a protective mechanism against bile acid–induced toxicity. Together, these findings indicate that PFAS-mediated modulation of LCA metabolism is a conserved response across species and experimental models, with potential relevance for developmental programming and long-term metabolic disease susceptibility.

We acknowledge some limitations of the study. As the exposure started on day 4, the model may impact PFOA on the organogenesis, which is largely finished by ED4. Our multiple-target PCR array was focused on lipid metabolism, so it does not cover the bile acid metabolism related pathways. However, our metabolic coverage on bile acid metabolism is broad, compensating the limitation of our targeted PCR panel. The strengths of the study include broad concentration range of exposure, and comprehensive metabolomics characterization of the liver related metabolites. By utilizing the in ovo injection model, we can directly assess the impact of environmental contaminants on the developing embryo without the confounding influences of maternal physiology, such as maternal metabolism, placental transfer, or lactation. This allows for a more precise understanding of the direct effects of environmental exposures on early development. Furthermore, by isolating these direct effects, we can focus on the specific developmental disruptions caused by the environmental factors we are studying, which might otherwise be masked by maternal influences in mammalian models.

In summary, our findings demonstrate that prenatal PFOA exposure induces selective and mechanistically coherent metabolic reprogramming in the embryonic liver — particularly affecting bile acid, lipid, and amino acid metabolism — in a manner consistent with early adaptive or stress-response signaling rather than overt toxicity. The strong and conserved modulation of lithocholic acid (LCA) and its conjugates across avian, murine, human fetal, and hepatocyte models suggests that PFAS-induced bile acid dysregulation may represent a fundamental and evolutionarily conserved mechanism of developmental interference. Given the central role of bile acids as metabolic signaling molecules and endocrine regulators, such alterations during critical developmental windows could have lasting effects on organ maturation, immune programming, and long-term metabolic disease susceptibility. These results therefore support growing concern that low-dose PFAS exposure during fetal development may contribute to adverse health trajectories through subtle but biologically meaningful disruption of hepatic signaling networks.

## Supplementary Information

Below is the link to the electronic supplementary material.

Supplementary Material 1

## Data Availability

Data will be made available on request.
