# Supplementary material for: Perfluorooctanoic acid disrupts hepatic metabolism in the developing chicken embryo
Source: Metabolomics. 2025 Dec 1;22(1):11. doi: 10.1007/s11306-025-02374-5 (PMC12669338; doi:10.1007/s11306-025-02374-5)
Supplement: Supplementary file 1 — Supplementary Material 1 [file 11306_2025_2374_MOESM1_ESM.docx]

**Supplementary material for**

**Perfluorooctanoic Acid Disrupts Hepatic Metabolism in the Developing Chicken Embryo**

Nikolai Scherbak^1^, Daniel Duberg^1^, Matilda Kråkström^2^, Pauli Tikka^2^, Alex M. Dickens^2,3^, Magnus Engwall^1^, Matej Orešič^2,4,5^, Tuulia Hyötyläinen^1,^*

^1^MTM Research Centre, School of Science and Technology, Örebro University, SE-701 82 Örebro, Sweden

^2^Turku Centre for Biotechnology, University of Turku and Åbo Akademi University, FI-20520 Turku, Finland

^3^Department of Chemistry, University of Turku, Turku, 20500, Finland

^4^School of Medical Sciences, Örebro University, SE-701 82 Örebro, Sweden

^5^Department of Life Technologies, University of Turku, Turku, FI-20014 Turku, Finland

*Correspondence:

Prof. Tuulia Hyötyläinen, Ph.D.

MTM Research Centre, School of Science and Technology, Örebro University, SE-701 82 Örebro, Sweden.

Email: [tuulia.hyotylainen@oru.se](mailto:tuulia.hyotylainen@oru.se)

Phone: +46 19 303487

**Table of contents**

**Supplementary methods**

**Supplementary figures and tables**

- **Supplementary Figure 1**. Impact of PFOA exposure on the weight of the liver.
- **Supplementary Table 1.** MRM settings used for steroid analysis.
- **Supplementary Table 2.** Metabolites showing significant association (LIMMA, adjusted with liver weight) with PFOA treatment.
- **Supplementary Table 3.** Results of mediation analysis, with bile acids as mediators and lipids as outcomes. Average Causal Mediated Effect (ACME), Average Direct Effect (ADE), corresponding p-values, and proportion of mediation are shown. Only results with significant ACME are shown. The indirect effects are graphically shown in **Figure 5**.

**Supplementary methods**

**Standards**

The lipid standards were from Avanti Polar Lipids Inc. (Alabaster, AL, USA). 13C-labeled PFAS internal standards (IS), 13C-labeled performance standards, and native calibration standards (perfluorocarboxylic acids (PFCAs) and perflurosulfonic acids (PFSAs)) were purchased from Wellington Laboratories (Guelph, Ontario, Canada). One native performance standard, 7H-dodecafluoroheptanoic acid, was purchased from ABCR (Karlsruhe, Germany). Chenodeoxycholic acid (CDCA), Cholic acid (CA), Deoxycholic acid (DCA), Glycochenodeoxycholic acid (GCDCA), Glycocholic acid (GCA), Glycodehydrocholic acid (GDCA), Glycohyocholic acid (GHCA) were obtained from Sigma-Aldrich (St. Luis, MO, USA). Glycohyodeoxycholic acid (GHDCA). Hyocholic acid (HCA), Hyodeoxycholic acid (HDCA), Litocholic acid (LCA), alpha-Muricholic acid (αMCA), Tauro-alpha-muricholic acid (T-α-MCA), Tauro-beta-muricholic acid(T-β-MCA), Taurochenodeoxycholic acid (TCDCA), Taurocholic acid (TCA), Taurodehydrocholic acid (THCA), Taurodeoxycholic acid (TDCA), Taurohyodeoxycholic acid (THDCA), Taurolitocholic acid (TLCA), Tauro-omega-muricholic acid (TωMCA) and Tauroursodeoxycholic acid (TUDCA) were from Steraloids (Newport, RI, U.S.A). Glycodeoxycholic acid (GDCA) and ursocholic acid (UDCA) were from Fluka (Buchs, Switzerland). Glycolitocholic acid (GLCA and Glycoursodeoxycholic acid (GUDCA) were from Calbiochem (Gibbstown, NJ, U.S.A). Internal standards CA-d4, LCA-d4, UDCA-d4, CDCA-d4, DCA-d4, GCA-d4, GLCA-d4, GUDCA-d4 and GCDCA-d4 were obtained from Qmx laboratories Ltd. (Essex, UK). For quality assurance (QA), standard reference material serum SRM 1950 (for lipidomics and metabolomics) and 1957 (for PFAS and bile acids) was purchased from the National Institute of Standards and Technology (NIST) at the US Department of Commerce (Washington, DC, USA). 2-diheptadecanoyl-sn-glycero-3-phosphoethanolamine (PE(17:0/17:0)), N-heptadecanoyl-D-erythro-sphingosylphosphorylcholine (SM(d18:1/17:0)), N-heptadecanoyl-D-erythro-sphingosine (Cer(d18:1/17:0)), 1,2-diheptadecanoyl-sn-glycero-3-phosphocholine (PC(17:0/17:0)), 1-heptadecanoyl-2-hydroxy-sn-glycero-3-phosphocholine (LPC(17:0)) and 1-palmitoyl-d31-2-oleoyl-sn-glycero-3-phosphocholine (PC(16:0/d31/18:1)), were purchased from Avanti Polar Lipids, Inc. (Alabaster, AL, USA), and, triheptadecanoylglycerol (TG(17:0/17:0/17:0)) and Cholesteryl 1-heptadecanoic acid (CE17:0) was purchased from Larodan AB (Solna, Sweden). Ultra purity water for the steroid analyses were obtained from Honeywell (Morris Plains, NJ, USA). Ammonium fluoride was obtained from Sigma-Aldrich (Saint Louis, MO, USA). 11-ketotestosterone, 11-keto-dihydrotestosterone and 11-ketotestosterone were obtained from Cayman Chemical (Ann Arbor, MI, USA); Androsteroneand Pregnenolone were obtained from Fisher Scientific (Waltham, MA, USA); 11-Deoxycorticosterone, were obtained from, Adrenosterone, Aldosterone, Androstenedione, Corticosterone, Cortisol, Cortisone, Dehydroepiandrosterone, Dihydrotestosterone, Estradiol, Estrone, Progesterone, Testosterone / Epi-testosterone were obtained from Sigma-Aldrich (Saint Louis, MO, USA); 11-deoxycortisol and 17α-OH-pregnenolone were obtained from Toronto Research Chemicals (North York, ON, Canada). Internal standards 11-ketotestosterone-d3 and 5a-dihydro-11-ketotestosterone-d3 were obtained from Cayman Chemicals (Ann Arbor, MI, USA); Testosterone-d3, Aldosterone-d7, Dihydrotestosterone-d4 were obtained from IsoSciences (Ambler, PA, USA); Androsterone-d4, 11β-OH-androstenedione-d4 were obtained from Sigma-Aldrich (Saint Louis, MO, USA); 17α-OH-pregnenolone, Corticosterone-d8, Pregnenolone-d4, 11-deoxy-cortisol-d7, 11-deoxycorticosterone-d7, Cortisol-d4, Progesterone-d9, Cortisone-d8, 17α-OH-pregnenolone -d8, Estradiol-d4, Estrone-d4 and Dehydroepiandrosterone-d2, Androstendione-d3 were obtained from Toronto Research Chemicals (North York, ON, Canada).

**LC-MS analysis**

The analytical conditions are shown in Supplementary Table 1.

**Supplementary Table 1.** LC-MS conditions for the three methods utilized in metabolomics analysis.

| Conditions | Polar/semipolar compounds | Lipidomics | Steroids |
| --- | --- | --- | --- |
| Injection volume | 10 µL | 1 µL | 10 µL |
| Column | C18 precolumn (Waters Corporation, Wexford, Ireland) and an inline filter, pore size 0.2 µm (Waters Corporation, Wexford, Ireland). + ACQUITY UPLC® BEH C18 column (2.1 mm × 100 mm, particle size 1.7 µm) by Waters (Milford, MA, USA) | C18 precolumn (Waters Corporation, Wexford, Ireland) and an inline filter, pore size 0,2 µm (Waters Corporation, Wexford, Ireland). + ACQUITY UPLC® BEH C18 column (2.1 mm × 100 mm, particle size 1.7 µm) by Waters (Milford, MA, USA) | Biphenyl precolumn (Phenomenex, Torrance, CA, USA) Kinetex 1.7 um Biphenyl 100 A LC Column 100 x 2.1 mm (Phenomenex, Torrance, CA, USA) |
| Mobile phases | A H_2_O:MeOH (v/v 70:30) with 2 mM ammonium acetate  B MeOH with containing 2 mM ammonium acetate | A 10 mM ammonium acetate and 0.1% Formic Acid in H_2_O  B Acetonitrile:Isopropanol (v/v 1:1) with 0.1% Formic Acid and 10 mM ammonium acetate | A H_2_O  B methanol  Post column infusion: 1 mM NH4F in water/methanol (10/90), 100 µLmin-1 |
| Gradient | - 0-1.5 min: B was increased from 5% to 30% - 1.5-4.5 min. - B increased to 70%; - 4.5-7.5 min. - B increased to 100% and held for 5.5 min. - A post-time of 6 min. | - 0-2 mi.n, B was increased from 35% to 80% - 2-7 min., B increased to 100% - 7-14 min., B was held at 100% - A post-time of 7 min. | - 0-3.5 min., B was increased from 38 to 65 % - 3.5-9.5 min., B was increased to 68 % - 9.5-13.5 min., B was increased to 76 % - 13.5-14.5 min., B was increased to 100 % - 14.5-19 min., B was held at 100 % - A post time of 2 min. |
| Flow rate | 0.4 mL/min | 0.4 mL/min | 0.3 mL/min |
| MS conditions | Dual ESI ionization source with capillary voltage 4.5 kV, nozzle voltage 1500 V, N2 pressure in the nebulized was 21 psi and the N2 flow rate and temperature as sheath gas was 11 Lmin-1 and 379 °C, respectively. Drying gas flow was set to 10 Lmin-1 and temperature to 150 °C. m/z range 80-1700 in negative ion mode. | Dual ESI ionization source with capillary voltage 3.64 kV, nozzle voltage 1500 V, N2 pressure in the nebulized was 21 psi and the N2 flow rate and temperature as sheath gas was 11 Lmin-1 and 379 °C, respectively. Drying gas flow was set to 10 Lmin-1 and temperature to 193 °C.  m/z range 100-3000 in positive ion mode. | OptiFlow Pro ionization source with source temperature 400 °C, spray voltage 1500 V, ion source gas 1 40 gsi, ion source gas 2 70 psi and entrance potential 10 V. Instrument was operated in positive and negative scheduled MRM mode. Transitions used are presented in **Supplementary Table 1.** |

**
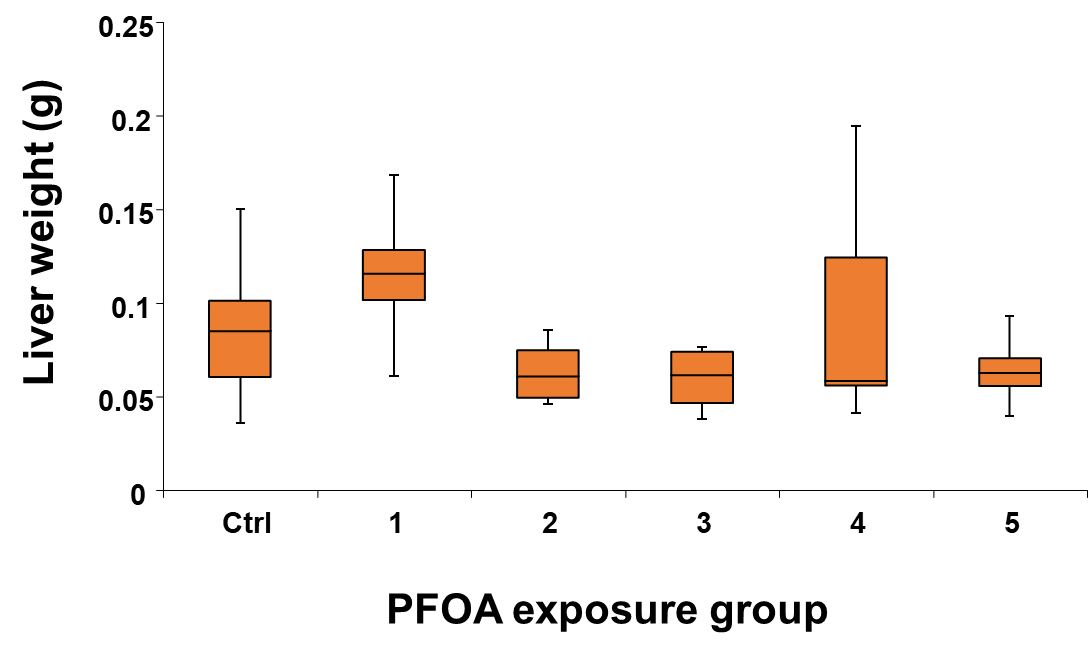
**

**Supplementary Figure 1.** Impact of PFOA exposure on the weight of the liver.

**Supplementary Table 2.** MRM settings used for steroid analysis.

| **MS/MS transition conditions** | | | | | | | | | | |  |
| --- | --- | --- | --- | --- | --- | --- | --- | --- | --- | --- | --- |
| Group | Compound ID | RT, min | RT window, (+/- s) | Q1 | Q2 | Dwell time, ms | CE, V | CXP, V | Q0 | ionization mode | LOQ  (pM) |
| F | d4-F | 5.572 | 45 | 367.3 | #### | 152.4 | 29 | 16 | -10 | pos | - |
| F | F 1 | 5.611 | 45 | 363.1 | #### | 141.5 | 31 | 8 | -10 | pos | 6.67 |
| F | F 2 | 5.601 | 45 | 363.1 | 91.0 | 143.2 | 83 | 10 | -10 | pos | 6.67 |
| E | d8-E | 6.166 | 45 | 369.2 | #### | 87.4 | 33 | 20 | -10 | pos | - |
| E | E 1 | 6.255 | 45 | 361.1 | #### | 81.3 | 31 | 26 | -10 | pos | 6.67 |
| E | E 2 | 6.245 | 45 | 361.1 | 77.0 | 81.8 | 107 | 10 | -10 | pos | 33.33 |
| 11-KT | d3-11-KT | 7.785 | 45 | 306.2 | #### | 95.2 | 31 | 26 | -10 | pos | - |
| 11-KT | 11-KT 1 | 7.823 | 45 | 303.4 | #### | 96.8 | 31 | 26 | -10 | pos | 6.67 |
| 11-KT | 11-KT 2 | 7.823 | 45 | 303.4 | #### | 96.8 | 31 | 0 | -10 | pos | 6.67 |
| 17a-OHP5 | d3-17a-OHP5 | 7.372 | 45 | 336.2 | #### | 58.2 | 15 | 17 | 10 | pos | - |
| 17a-OHP5 | 17a-OHP5 1 | 7.408 | 45 | 333.1 | #### | 59.0 | 13 | 22 | 10 | pos | 666.67 |
| 17a-OHP5 | 17a-OHP5 2 | 7.408 | 45 | 333.5 | #### | 59.0 | 10 | 18 | 10 | pos | 666.67 |
| S | d7-S | 8.874 | 45 | 354.1 | #### | 3.0 | 33 | 16 | -10 | pos | - |
| S | S 1 | 8.994 | 45 | 347.1 | 97.0 | 3.0 | 27 | 12 | -10 | pos | 6.67 |
| S | S 2 | 8.994 | 45 | 347.1 | #### | 3.0 | 33 | 16 | -10 | pos | 6.67 |
| B | d8-B | 9.98 | 45 | 355.5 | #### | 33.8 | 32 | 11 | -10 | pos | - |
| B | B 1 | 9.978 | 45 | 347.5 | #### | 33.8 | 21 | 22 | -10 | pos | 33.33 |
| B | B 2 | 9.965 | 45 | 347.1 | #### | 34.2 | 29 | 8 | -10 | pos | 33.33 |
| 11b-OHA4 | d4-11b-OHA4 | 9.297 | 45 | 307.3 | #### | 64.1 | 25 | 15 | -10 | pos | - |
| 11b-OHA4 | 11b-OHA4 1 | 9.401 | 45 | 303.2 | #### | 53.9 | 24 | 14 | -10 | pos | 6.67 |
| 11b-OHA4 | 11b-OHA4 2 | 9.386 | 45 | 303.2 | #### | 55.6 | 22 | 16 | -10 | pos | 6.67 |
| 11-KDHT | d3-11-KDHT | 9.283 | 45 | 308.4 | #### | 100.0 | 25 | 24 | 50 | pos | - |
| 11-KDHT | 11-KDHT 1 | 9.268 | 45 | 305.4 | #### | 100.0 | 25 | 21 | 50 | pos | 13.33 |
| 11-KDHT | 11-KDHT 2 | 9.197 | 45 | 305.4 | #### | 71.8 | 23 | 20 | 50 | pos | 13.33 |
| 11-KA4 | 11-KA4 1 | 9.588 | 45 | 301.2 | #### | 100.0 | 31 | 17 | -10 | pos | 3.33 |
| 11-KA4 | 11-KA4 2 | 9.588 | 45 | 301.2 | #### | 65.4 | 31 | 15 | -10 | pos | 3.33 |
| DHEA | DHEA 1 | 10.841 | 45 | 289.1 | #### | 62.0 | 15 | 46 | 30 | pos | 3.33 |
| DHEA | DHEA 2 | 10.854 | 45 | 289.4 | #### | 62.4 | 11 | 28 | 30 | pos | 3.33 |
| DHEA | d2-DHEA 1 | 10.79 | 45 | 291.2 | #### | 51.6 | 16 | 14 | 30 | pos | - |
| DHEA | d2-DHEA 2 | 10.79 | 45 | 291.2 | #### | 51.6 | 12 | 15 | 30 | pos | - |
| T/Epi-T | d3-T | 11.837 | 45 | 292.1 | 97.0 | 76.5 | 29 | 12 | -10 | pos | 3.33 |
| T/Epi-T | T/Epi-T 1 | 11.896 | 45 | 289.1 | 97.0 | 74.6 | 29 | 12 | -10 | pos | 3.33 |
| T/Epi-T | T/Epi-T 2 | 11.912 | 45 | 289.1 | #### | 74.1 | 31 | 6 | -10 | pos | 3.33 |
| 17aOH-P4 | d8-17aOH-P4 | 11.95 | 45 | 339.1 | #### | 73.6 | 27 | 12 | -10 | pos | - |
| 17aOH-P4 | 17aOH-P4 1 | 12.124 | 45 | 331.1 | #### | 73.5 | 31 | 12 | -10 | pos | 6.67 |
| 17aOH-P4 | 17aOH-P4 2 | 12.13 | 45 | 331.1 | 96.9 | 73.7 | 29 | 12 | -10 | pos | 6.67 |
| AN | d4-AN | 12.533 | 45 | 277.2 | #### | 79.7 | 19 | 25 | -10 | pos | - |
| AN | AN 1 | 12.62 | 45 | 273.0 | #### | 82.1 | 20 | 12 | -10 | pos | 66.67 |
| AN | AN 2 | 12.617 | 45 | 273.0 | #### | 82.0 | 30 | 14 | -10 | pos | 66.67 |
| DHT | d4-DHT | 13.129 | 45 | 295.2 | #### | 130.5 | 23 | 14 | 10 | pos | - |
| DHT | DHT 1 | 13.274 | 45 | 291.3 | #### | 140.6 | 21 | 30 | 10 | pos | 133.33 |
| DHT | DHT 2 | 13.247 | 45 | 291.4 | #### | 138.5 | 10 | 18 | 10 | pos | 133.33 |
| A4 | d3-A4 1 | 14.711 | 45 | 290.2 | #### | 5.0 | 29 | 16 | -10 | pos | - |
| A4 | d3-A4 2 | 14.711 | 45 | 290.2 | #### | 5.0 | 32 | 14 | -10 | pos | - |
| A4 | A4 1 | 14.757 | 45 | 287.0 | 97.0 | 5.0 | 27 | 14 | -10 | pos | 6.67 |
| A4 | A4 2 | 14.769 | 45 | 287.0 | 78.9 | 5.0 | 67 | 10 | -10 | pos | 6.67 |
| DOC | d7-DOC 1 | 14.942 | 45 | 338.2 | #### | 83.5 | 28 | 11 | -10 | pos | - |
| DOC | d7-DOC 2 | 14.931 | 45 | 338.2 | #### | 83.9 | 31 | 20 | -10 | pos | - |
| DOC | DOC 1 | 15.012 | 45 | 331.2 | 97.0 | 84.0 | 29 | 16 | -10 | pos | 6.67 |
| DOC | DOC 2 | 15.012 | 45 | 331.2 | #### | 84.0 | 31 | 12 | -10 | pos | 6.67 |
| P5 | d4-P5 | 14.595 | 45 | 321.1 | #### | 106.4 | 20 | 16 | -10 | pos | - |
| P5 | P5 1 | 14.688 | 45 | 317.5 | #### | 99.1 | 15 | 16 | -10 | pos | 333.33 |
| P5 | P5 2 | 14.711 | 45 | 317.1 | #### | 99.1 | 17 | 16 | -10 | pos | 333.33 |
| P4 | d9-P4 | 15.9 | 45 | 324.0 | #### | 157.7 | 23 | 10 | -10 | pos | - |
| P4 | P4 1 | 15.943 | 45 | 315.1 | 97.1 | 168.6 | 23 | 10 | -10 | pos | 6.67 |
| P4 | P4 2 | 15.922 | 45 | 315.1 | #### | 160.1 | 27 | 10 | -10 | pos | 6.67 |
| A | d7-A | 7.23 | 45 | 366.2 | #### | 59.3 | -26 | -21 | 10 | neg | - |
| A | A 1 | 7.39 | 45 | 359.1 | #### | 58.5 | -22 | -35 | 10 | neg | 33.33 |
| A | A 2 | 7.39 | 45 | 359.1 | #### | 58.5 | -26 | -21 | 10 | neg | 33.33 |
| E2 | d4-E2 | 6.75 | 45 | 275.0 | #### | 62.9 | -55 | -22 | 10 | neg | - |
| E2 | E2 1 | 6.78 | 45 | 271.0 | #### | 62.2 | -52 | -19 | 10 | neg | 33.33 |
| E2 | E2 2 | 6.8 | 45 | 271.0 | #### | 62.1 | -60 | -21 | 10 | neg | 33.33 |
| E1 | d4-E1 | 10.26 | 45 | 273.0 | #### | 35.1 | -60 | -20 | 10 | neg | - |
| E1 | E1 1 | 10.36 | 45 | 269.1 | #### | 36.5 | -48 | -15 | 10 | neg | 6.67 |
| E1 | E1 2 | 10.35 | 45 | 269.1 | #### | 36.5 | -70 | -15 | 10 | neg | 6.67 |

**Supplementary Table 3.** Recoveries of the internal standards in the liver extract.

| **ISTD** | **recovery %** |
| --- | --- |
| ISTD-PFHxS-18O2 | 93.24 |
| ISTD-PFOS-13C4 | 91.20 |
| ISTD-PFUNdA-13C2 | 75.72 |
| ISTD-PFOA-13C4 | 75.31 |
| ISTD-PFNA-13C5 | 91.08 |
| ISTD-CA-d4 | 83.5 |
| ISTD-CDCA-d4 | 65.38 |
| ISTD-DCA-d4 | 79.58 |
| ISTD-GCA-d4 | 89.84 |
| ISTD-GDCA-d4 | 90.73 |
| ISTD-GLCA-d4 | 91.15 |
| ISTD-GUDCA-d4 | 88.19 |
| ISTD-HDCA-d4 | 68.67 |
| ISTD-LCA-d4 | 92.65 |
| ISTD-TCA-d4 | 44.57 |
| ISTD-UDCA-d4 | 82.84 |
| ISTD-Tryphtophan-d5 | 73.42 |
| ISTD-Heptadecanoic acid | 131.22 |

**Supplementary Table 4.** Metabolites showing significant association (LIMMA, adjusted with liver weight) with PFOA treatment. The ionization mode, adduct m/z value and Δppm are also shown

| **Metabolite class** | **Metabolite** | **Mode** | **Ion/Adduct** | **mz** | **Δppm** | **t score** | **p-value** | **adj. p-value** |
| --- | --- | --- | --- | --- | --- | --- | --- | --- |
| Amino acid | 5-Oxoproline | ESI- | [M-H]- | 128.0351 | 2.4 | 2.53 | 0.0115 | 0.0459 |
| Amino acid | Aspartic acid | ESI- | [M-H]- | 132.0304 | 5.5 | 3.08 | 2.07E-03 | 0.015 |
| Amino acid | Glutamic acid | ESI- | [M-H]- | 146.0457 | 2.5 | 2.49 | 0.0128 | 0.0494 |
| Amino acid | Leucine/Isoleucine | ESI- | [M-H]- | 130.0872 | 3.3 | 3.52 | 4.36E-04 | 0.0069 |
| Amino acid | Phenylalanine | ESI- | [M-H]- | 164.0712 | 0.4 | 2.84 | 4.49E-03 | 0.0257 |
| Amino acid | Proline | ESI- | [M-H]- | 114.0557 | 1.9 | 3.56 | 3.78E-04 | 0.0066 |
| Amino acid | Serine | ESI- | [M-H]- | 104.0357 | 8.8 | 3.52 | 4.31E-04 | 0.0069 |
| Amino acid | Threonine | ESI- | [M-H]- | 118.0508 | 3.8 | 4.19 | 2.76E-05 | 1.22E-03 |
| Amino acid | tyrosine | ESI- | [M-H]- | 180.0664 | 1.7 | 3.36 | 7.71E-04 | 0.0083 |
| Amino acid | Valine | ESI- | [M-H]- | 116.0712 | 0.7 | 3.44 | 5.84E-04 | 0.0073 |
| Bile acid | CDCA | ESI- | [M-H]- | 391.2852 | 0.9 | 3.82 | 1.33E-04 | 3.20E-03 |
| Bile acid | GCDCA | ESI- | [M-H]- | 448.3068 | 1.2 | -3.04 | 2.34E-03 | 0.0167 |
| Bile acid | TCDCA | ESI- | [M-H]- | 498.2902 | 2.5 | 3.8 | 1.44E-04 | 3.20E-03 |
| Bile acid | THDCA | ESI- | [M-H]- | 498.2879 | -2.0 | 4.57 | 4.92E-06 | 2.80E-04 |
| Bile acid | TLCA | ESI- | [M-H]- | 482.2948 | 1.7 | 4.94 | 7.87E-07 | 7.83E-05 |
| Bile acid | TLCA isomer | ESI- | [M-H]- | 482.2948 | 1.7 | 5.06 | 4.31E-07 | 7.32E-05 |
| Bile acid | TMCA | ESI- | [M-H]- | 514.2828 | -2.0 | 3.21 | 1.31E-03 | 0.0114 |
| Fatty acid | C20:5 | ESI- | [M-H]- | 301.2164 | -1.1 | 4.46 | 8.12E-06 | 4.04E-04 |
| Fatty acid | Hexanoic acid | ESI- | [M-H]- | 115.0763 | 3.2 | 2.51 | 0.0119 | 0.0471 |
| Fatty acid | Octanoic acid | ESI- | [M-H]- | 143.1076 | 3.0 | 2.87 | 4.13E-03 | 0.0241 |
| Fatty acid | stearic acid | ESI- | [M-H]- | 283.2651 | 5.1 | 2.87 | 4.05E-03 | 0.0241 |
| Lipid | DG(14:0/20:1) | ESI+ | [M+NH4]+ | 612.5554 | -1.2 | 2.78 | 5.43E-03 | 0.0292 |
| Lipid | DG(14:0/22:5) | ESI+ | [M+NH4]+ | 632.5238 | -1.6 | 4.7 | 2.64E-06 | 0.0014 |
| Lipid | DG(16:1/18:1) | ESI+ | [M+NH4]+ | 610.5393 | -1.9 | 2.6 | 9.23E-03 | 0.0408 |
| Lipid | DG(16:1/20:1) | ESI+ | [M+NH4]+ | 638.5712 | -1.0 | 3.25 | 1.16E-03 | 0.0104 |
| Lipid | DG(18:0/20:5) | ESI+ | [M+NH4]+ | 660.5553 | -1.2 | 3.14 | 1.68E-03 | 0.0128 |
| Lipid | DG(18:1/20:2) | ESI+ | [M+NH4]+ | 664.5863 | -1.6 | 2.98 | 2.90E-03 | 0.0189 |
| Lipid | DG(20:5/22:6) | ESI+ | [M+NH4]+ | 704.5225 | -3.3 | 2.65 | 8.12E-03 | 0.0367 |
| Lipid | DG(36:3) | ESI+ | [M+NH4]+ | 636.555 | -1.8 | 3.65 | 2.59E-04 | 0.0052 |
| Lipid | DG(40:4) | ESI+ | [M+NH4]+ | 690.6022 | -1.3 | 2.59 | 9.48E-03 | 0.041 |
| Lipid | DG(44:10) | ESI+ | [M+NH4]+ | 734.5703 | -2.0 | 2.92 | 3.52E-03 | 0.0216 |
| Lipid | Dodecanoylcarnitine | ESI+ | [M+H]+ | 314.2311 | -6.4 | -4.13 | 3.57E-05 | 0.0041 |
| Lipid | Hexadecenoylcarnitine | ESI+ | [M-H]- | 398.3253 | -4.5 | -3.15 | 1.65E-03 | 0.0128 |
| Lipid | LPC(14:0) | ESI+ | [M+H]+ | 468.3072 | -2.8 | 2.75 | 5.96E-03 | 0.0312 |
| Lipid | LPC(18:1) | ESI+ | [M+H]+ | 522.3545 | -1.8 | 2.55 | 0.0108 | 0.0454 |
| Lipid | LPC(18:2) | ESI+ | [M+H]+ | 520.3382 | -3.1 | 3.88 | 1.05E-04 | 2.78E-03 |
| Lipid | LPC(20:3) | ESI+ | [M+H]+ | 546.3542 | -2.2 | 3.08 | 2.06E-03 | 0.015 |
| Lipid | LPC(20:4) | ESI+ | [M+H]+ | 544.3389 | -1.7 | 3.37 | 7.47E-04 | 0.0083 |
| Lipid | LPC(20:5) | ESI+ | [M+H]+ | 542.3211 | -5.6 | 3.33 | 8.76E-04 | 0.0089 |
| Lipid | LPE(20:4) | ESI+ | [M+H]+ | 502.292 | -1.5 | 3.35 | 7.99E-04 | 0.0084 |
| Lipid | LysoPE(18:1) | ESI+ | [M+H]+ | 480.3076 | -1.9 | 3.12 | 1.84E-03 | 0.0138 |
| Lipid | Myristoyl-L-carnitine | ESI+ | [M+H]+ | 372.3099 | -4.0 | -4.02 | 5.90E-05 | 0.0037 |
| Lipid | Octadecadienylcarnitine | ESI+ | [M+H]+ | 424.3421 | -0.1 | -2.68 | 7.41E-03 | 0.0347 |
| Lipid | PC(18:0/18:0) | ESI+ | [M+H]+ | 790.6328 | 1.0 | 3.27 | 1.08E-03 | 0.0101 |
| Lipid | PC(31:0) | ESI+ | [M+H]+ | 720.5535 | -0.4 | 3.31 | 9.37E-04 | 0.0093 |
| Lipid | PC(32:1) | ESI+ | [M+H]+ | 732.5525 | -1.8 | 2.69 | 7.12E-03 | 0.0341 |
| Lipid | PC(32:2) | ESI+ | [M+H]+ | 730.5375 | -0.8 | 2.96 | 3.09E-03 | 0.0195 |
| Lipid | PC(33:1) | ESI+ | [M+H]+ | 746.5688 | -0.7 | 3.76 | 1.73E-04 | 0.0036 |
| Lipid | PC(34:2(OH) | ESI+ | [M+H]+ | 774.5623 | -9.0 | 3.19 | 1.45E-03 | 0.0123 |
| Lipid | PC(34:3) | ESI+ | [M+H]+ | 756.5531 | -0.9 | 3.41 | 6.46E-04 | 0.0078 |
| Lipid | PC(34:3) | ESI+ | [M+H]+ | 756.5531 | -0.9 | 3.4 | 6.79E-04 | 0.008 |
| Lipid | PC(35:1) | ESI+ | [M+H]+ | 774.5997 | -1.3 | 3.8 | 1.45E-04 | 3.20E-03 |
| Lipid | PC(35:2) | ESI+ | [M+H]+ | 772.585 | -0.1 | 3.46 | 5.45E-04 | 0.0072 |
| Lipid | PC(35:3) | ESI+ | [M+H]+ | 770.5692 | -0.3 | 2.84 | 4.52E-03 | 0.0257 |
| Lipid | PC(35:4) | ESI+ | [M+H]+ | 768.552 | -2.4 | 4.12 | 3.83E-05 | 1.24E-03 |
| Lipid | PC(36:2) | ESI+ | [M+H]+ | 786.602 | 1.6 | 3.37 | 7.65E-04 | 0.0083 |
| Lipid | PC(36:3) | ESI+ | [M+H]+ | 784.5843 | -1.0 | 3.5 | 4.71E-04 | 0.0069 |
| Lipid | PC(36:3) | ESI+ | [M+H]+ | 784.5838 | -1.6 | 2.61 | 9.19E-03 | 0.0408 |
| Lipid | PC(36:3) | ESI+ | [M+H]+ | 784.5842 | -1.2 | 3.17 | 1.55E-03 | 0.0126 |
| Lipid | PC(36:4) | ESI+ | [M+H]+ | 782.5666 | -3.5 | 4.12 | 3.75E-05 | 1.24E-03 |
| Lipid | PC(36:5) | ESI+ | [M+H]+ | 780.5535 | -0.4 | 5.01 | 5.52E-07 | 7.32E-05 |
| Lipid | PC(37:2) | ESI+ | [M+H]+ | 800.6162 | -0.3 | 2.58 | 9.97E-03 | 0.0426 |
| Lipid | PC(37:2) | ESI+ | [M+H]+ | 800.6159 | -0.7 | 2.68 | 7.38E-03 | 0.0347 |
| Lipid | PC(37:4) | ESI+ | [M+H]+ | 796.5855 | 0.5 | 3.56 | 3.80E-04 | 0.0066 |
| Lipid | PC(38:1) | ESI+ | [M+H]+ | 816.6478 | 0.2 | 3.03 | 2.49E-03 | 0.0174 |
| Lipid | PC(38:2) | ESI+ | [M+H]+ | 814.6319 | -0.2 | 2.7 | 6.97E-03 | 0.0338 |
| Lipid | PC(38:5) | ESI+ | [M+H]+ | 808.5843 | -1.0 | 4.11 | 3.96E-05 | 1.24E-03 |
| Lipid | PC(38:6) | ESI+ | [M+H]+ | 806.5702 | 1.0 | 3.48 | 5.04E-04 | 0.0069 |
| Lipid | PC(40:8) | ESI+ | [M+H]+ | 830.57 | 0.7 | 3 | 2.68E-03 | 0.0181 |
| Lipid | PE(16:0/18:1) | ESI+ | [M+H]+ | 718.538 | -0.1 | 2.73 | 6.36E-03 | 0.0325 |
| Lipid | PE(16:0/18:2) | ESI+ | [M+H]+ | 716.5227 | 0.3 | 4.62 | 3.84E-06 | 2.54E-04 |
| Lipid | PE(16:0/20:4) | ESI+ | [M+H]+ | 740.5227 | 0.3 | 2.95 | 3.16E-03 | 0.0196 |
| Lipid | PE(16:0/20:4) | ESI+ | [M+H]+ | 740.5245 | 2.7 | 2.66 | 7.73E-03 | 0.0358 |
| Lipid | PE(16:0/22:6) | ESI+ | [M+H]+ | 764.5242 | 2.2 | 2.76 | 5.80E-03 | 0.0308 |
| Lipid | PE(18:0/18:1) | ESI+ | [M+H]+ | 746.5695 | 0.1 | 4.11 | 4.05E-05 | 1.24E-03 |
| Lipid | PE(18:0/22:6) | ESI+ | [M+H]+ | 792.5541 | 0.3 | 2.71 | 6.72E-03 | 0.0337 |
| Lipid | PE(18:1e/22:4) | ESI+ | [M+H]+ | 780.5901 | -0.1 | 2.74 | 6.23E-03 | 0.0322 |
| Lipid | PE(18:1e/22:6) | ESI+ | [M+H]+ | 776.5597 | 1.1 | 3.51 | 4.55E-04 | 0.0069 |
| Lipid | PE(20:5/16:0) | ESI+ | [M+H]+ | 738.508 | 1.7 | 5.08 | 3.80E-07 | 7.32E-05 |
| Lipid | PE(38:5) | ESI+ | [M+H]+ | 766.5375 | -0.8 | 2.7 | 6.89E-03 | 0.0338 |
| Lipid | PE(O-38:5) or PE(P-38:4) | ESI+ | [M+H]+ | 752.5585 | -0.5 | 2.55 | 0.0108 | 0.0454 |
| Lipid | PE(P-16:0/22:6) | ESI+ | [M+H]+ | 748.5276 | 0.0 | 3.44 | 5.75E-04 | 0.0073 |
| Lipid | PE(P-18:0/18:1) | ESI+ | [M+H]+ | 730.5755 | 1.3 | 2.53 | 0.0114 | 0.0459 |
| Lipid | PE(P-18:0/18:2) | ESI+ | [M+H]+ | 728.5599 | 1.4 | 2.53 | 0.0113 | 0.0459 |
| Lipid | PE(P-18:0/22:5) + PE(P-20:1/20:4) | ESI+ | [M+H]+ | 778.5744 | -0.2 | 2.82 | 4.86E-03 | 0.0269 |
| Lipid | PE(P-18:0/22:5) + PE(P-20:1/20:4) | ESI+ | [M+H]+ | 778.5748 | 0.4 | 3.25 | 1.17E-03 | 0.0104 |
| Lipid | PG(34:2) | ESI+ | [M+H]+ | 747.5167 | -0.6 | 4.42 | 9.67E-06 | 0.0034 |
| Lipid | PG(38:5) | ESI+ | [M+H]+ | 797.5297 | -3.8 | 4.74 | 2.15E-06 | 0.0014 |
| Lipid | PG(40:6) | ESI+ | [M+H]+ | 823.5443 | -5.0 | 2.6 | 9.39E-03 | 0.041 |
| Lipid | TG(18:2/18:1/18:1) | ESI+ | [M+NH4]+ | 900.8003 | -1.4 | 2.71 | 6.77E-03 | 0.0337 |
| Lipid | TG(46:0) | ESI+ | [M+NH4]+ | 796.7379 | -1.3 | 3.15 | 1.65E-03 | 0.0128 |
| Lipid | TG(49:0) | ESI+ | [M+NH4]+ | 838.7852 | -0.7 | 3.3 | 9.87E-04 | 0.0096 |
| Lipid | TG(P-52:1) | ESI+ | [M+NH4]+ | 864.8372 | -0.8 | -2.5 | 0.0125 | 0.0489 |
| Other | Cholecalciferol (D3) | ESI+ | [M+H]+ | 385.3451 | -3.5 | 2.53 | 0.0113 | 0.0459 |
| Other | D-Glyceric acid | ESI- | [M-H]- | 105.019 | 2.3 | 3.17 | 1.51E-03 | 0.0125 |
| Other | Gallic acid | ESI- | [M-H]- | 169.0127 | -9.2 | 2.9 | 3.73E-03 | 0.0225 |
| Other | Glycerol-3-phosphate | ESI- | [M-H]- | 171.0064 | 3.1 | 2.65 | 8.00E-03 | 0.0366 |
| Other | Guanosine | ESI- | [M-H]- | 282.0839 | 0.1 | 3.02 | 2.54E-03 | 0.0174 |
| Other | Hypoxanthine | ESI- | [M-H]- | 135.0312 | 3.7 | 3.27 | 1.09E-03 | 0.0101 |
| Other | Itaconic acid | ESI- | [M-H]- | 129.0205 | 8.8 | 3.48 | 5.01E-04 | 0.0069 |
| Other | Succinic acid | ESI- | [M-H]- | 117.0189 | 1.3 | 2.8 | 5.08E-03 | 0.0277 |

**Supplementary Table 5.** Results of mediation analysis, with bile acids as mediators and lipids as outcomes. Average Causal Mediated Effect (ACME), Average Direct Effect (ADE), corresponding p-values, and proportion of mediation are shown. Only results with significant ACME are shown. The indirect effects are graphically shown in **Figure 5**.

| **Contaminant >> Bile Acid >> Lipid** | **ACME** | **P(ACME)** | **ADE** | **P(ADE)** | **Proportion Mediated** |
| --- | --- | --- | --- | --- | --- |
| PFOA_L TLCA LPE | 0.916 | <0.001 | -0.321 | 0.304 | **1.535** |
| PFOA_L TLCA LPC | 1.000 | <0.001 | -0.607 | 0.094 | **2.484** |
| PFOA_L TCDCA SM | 0.571 | <0.001 | -0.041 | 0.834 | **1.074** |
| PFOA_L TCDCA PEO/P | 0.583 | <0.001 | 0.037 | 0.814 | **0.933** |
| PFOA_L TCDCA PE | 0.457 | <0.001 | 0.337 | 0.018 | **0.573** |
| PFOA_L TCDCA PC_O/P | 0.833 | <0.001 | -0.621 | 0.002 | **2.988** |
| PFOA_L TCDCA PC | 0.560 | <0.001 | 0.000 | 0.996 | **1.000** |
| PFOA_L TCDCA LPE | 0.733 | <0.001 | -0.136 | 0.362 | **1.229** |
| PFOA_L TCDCA LPC | 0.821 | <0.001 | -0.436 | 0.016 | **2.090** |
| PFOA_L TCDCA HexCer | 0.803 | <0.001 | -0.619 | 0.002 | **2.937** |
| PFOA_L TCDCA CER | 0.757 | <0.001 | -0.577 | 0.016 | **2.868** |
| PFOA_L Tauro-muricholic acid SM | 0.594 | <0.001 | -0.068 | 0.728 | **1.113** |
| PFOA_L Tauro-muricholic acid PE | 0.338 | <0.001 | 0.450 | 0.000 | 0.430 |
| PFOA_L TLCA HexCer | 0.968 | 0.002 | -0.783 | 0.052 | **3.295** |
| PFOA_L Tauro-muricholic acid DG | 0.278 | 0.002 | 0.569 | 0.000 | 0.321 |
| PFOA_L GCDCA SM | 0.335 | 0.002 | 0.197 | 0.292 | **0.629** |
| PFOA_L Tauro-muricholic acid CAR | 0.498 | 0.004 | -0.631 | 0.016 | -1.548 |
| PFOA_L GCDCA LPE | 0.341 | 0.004 | 0.261 | 0.160 | **0.569** |
| PFOA_L TCDCA TG_O | 0.570 | 0.006 | -0.672 | 0.016 | -1.654 |
| PFOA_L Tauro-muricholic acid LPE | 0.415 | 0.006 | 0.175 | 0.406 | **0.707** |
| PFOA_L Tauro-muricholic acid CER | 0.502 | 0.006 | -0.304 | 0.250 | **1.715** |
| PFOA_L GCDCA LPC | 0.347 | 0.006 | 0.053 | 0.802 | **0.858** |
| PFOA_L GCDCA CAR | 0.388 | 0.006 | -0.512 | 0.032 | -1.187 |
| PFOA_L TLCAisomer CE | 1.019 | 0.010 | -0.880 | 0.034 | **3.659** |
| PFOA_L THDCA DG | 0.390 | 0.010 | 0.455 | 0.018 | 0.456 |
| PFOA_L TCDCA DG | 0.293 | 0.010 | 0.557 | 0.000 | 0.347 |
| PFOA_L TCDCA CE | 0.503 | 0.018 | -0.360 | 0.178 | **1.766** |
| PFOA_L TCDCA CAR | 0.444 | 0.030 | -0.572 | 0.028 | -1.377 |
| PFOA_L TLCA PC_O/P | 0.829 | 0.032 | -0.616 | 0.118 | **2.692** |
| PFOA_L GCDCA PC_O/P | 0.321 | 0.032 | -0.116 | 0.578 | **1.171** |
| PFOA_L TLCA PE | 0.469 | 0.034 | 0.323 | 0.222 | **0.574** |
| PFOA_L TLCA CER | 0.826 | 0.034 | -0.639 | 0.112 | **2.798** |
| PFOA_L Tauro-muricholic acid PC_O/P | 0.398 | 0.036 | -0.185 | 0.508 | **1.298** |
| PFOA_L TbMCA PE | 0.142 | 0.042 | 0.648 | 0.000 | 0.173 |
| PFOA_L TLCAisomer HexCer | 0.731 | 0.044 | -0.559 | 0.192 | **2.482** |
| PFOA_L GCDCA DG | 0.167 | 0.044 | 0.686 | 0.000 | 0.185 |
| PFOA_L TbMCA PC_O/P | 0.308 | 0.046 | -0.098 | 0.436 | **1.182** |
